# Multiple Forms of Neural Cell Death in the Cyclical Brain Degeneration of A Colonial Chordate

**DOI:** 10.3390/cells12071041

**Published:** 2023-03-29

**Authors:** Chiara Anselmi, Federico Caicci, Tommaso Bocci, Matteo Guidetti, Alberto Priori, Veronica Giusti, Tom Levy, Tal Raveh, Ayelet Voskoboynik, Irving L. Weissman, Lucia Manni

**Affiliations:** 1 Hopkins Marine Station, Institute for Stem Cell Biology and Regenerative Medicine, Stanford University, Pacific Grove, CA 93950, USA; 2 Institute for Stem Cell Biology and Regenerative Medicine, Stanford University School of Medicine, Stanford, CA 94305, USA; 3 Wu Tsai Neurosciences Institute, Stanford University, Stanford, CA 94305, USA; 4 Dipartimento di Biologia, Università degli Studi di Padova, 35131 Padova, Italy; 5 “Aldo Ravelli” Center for Neurotechnology and Experimental Brain Therapeutics, Department of Health Sciences, University of Milan, 20142 Milan, Italy; 6 Department of Electronics, Information and Bioengineering, Politecnico di Milano, 20133 Milan, Italy; 7 San Camillo Hospital srl, IRCCS, 30126 Venezia, Italy; 8 Chan Zuckerberg Biohub, San Francisco, CA 94158, USA; 9 Department of Pathology, Stanford University School of Medicine, Stanford, CA 94305, USA

**Keywords:** apoptosis, autophagy, colonial chordate, lysosomal cell death, necroptosis

## Abstract

Human neuronal loss occurs through different cellular mechanisms, mainly studied in vitro. Here, we characterized neuronal death in *B. schlosseri*, a marine colonial tunicate that shares substantial genomic homology with mammals and has a life history in which controlled neurodegeneration happens simultaneously in the brains of adult zooids during a cyclical phase named takeover. Using an ultrastructural and transcriptomic approach, we described neuronal death forms in adult zooids before and during the takeover phase while comparing adult zooids in takeover with their buds where brains are refining their structure. At takeover, we found in neurons clear morphologic signs of apoptosis (i.e., chromatin condensation, lobed nuclei), necrosis (swollen cytoplasm) and autophagy (autophagosomes, autolysosomes and degradative multilamellar bodies). These results were confirmed by transcriptomic analyses that highlighted the specific genes involved in these cell death pathways. Moreover, the presence of tubulovesicular structures in the brain medulla alongside the over-expression of prion disease genes in late cycle suggested a cell-to-cell, prion-like propagation recalling the conformational disorders typical of some human neurodegenerative diseases. We suggest that improved understanding of how neuronal alterations are regulated in the repeated degeneration–regeneration program of *B. schlosseri* may yield mechanistic insights relevant to the study of human neurodegenerative diseases.

## 1. Introduction

The homeostatic maintenance of most organs and tissues is ensured throughout the life of an organism by a controlled balance of cell division and cell death. In vertebrates, the nervous system’s development involves both processes during the generation of functional circuitry [1]. Once the mature nervous system is established, the threshold required to induce cell death becomes much higher, and neuronal death is then limited to homeostasis maintenance [2]. Indeed, uncontrolled cellular proliferation can result in the development of disease such as cancer, whereas an excessive level of cell death is a manifestation of diseases such as Alzheimer’s and Parkinson’s [3]. Understanding how nervous system cell components preserve their equilibrium with respect to their environment is of great relevance to human-focused studies and clinical applications designed to investigate and mitigate the loss of brain functional abilities associated with aging and neurodegenerative diseases.

Today, studies of neuronal cell death are mainly performed in vitro using mammalian cell lines because of animal anatomical complexity. Reliable and simple animal models are very useful to in vivo studies on neuronal cell death and its role in the organism as a whole. Tunicates are marine invertebrates [4] considered the sister group of vertebrates [5]. More specifically, the marine colonial tunicate *Botryllus schlosseri*, which shares high genomic homology with mammals [6], can shed light on the evolution of neuronal cell death mechanisms relevant to aging and neurodegenerative diseases [7]. Tunicates, as translational models, are of key importance for two main reasons: (1) they provide an opportunity for studying the entire process of neurodegeneration, including those relevant to pre-clinical stages of human neurodegenerative disorders, when pharmacological interventions may still be effective; (2) in colonial species such as *B. schlosseri*, they provide a unique, combined model of cyclical degeneration and neural development, revealing possible mechanisms of neural protection in growing buds [7].

Three major types of cell death, conserved throughout evolution, have been described on the basis of their morphological manifestations: apoptosis, autophagy, and necrosis [3,4,5,6,7,8]. Each of them is characterized by the expression of specific and common gene pathways [1]. The term apoptosis describes a controlled (programmed) process of cell death marked by chromatin condensation, nuclear fragmentation, and the initial maintenance of the plasma membrane, followed by cell fragmentation into small vesicles [4]. During this process, several genes are selectively activated and participate in degenerative events. Phagocytes often remove apoptotic cells before they fragment, resulting in the containment of the dying cells within a tissue while reducing the risk of collateral damage to surrounding cells. The term necrosis initially referred to a poorly controlled, unregulated process, induced by external injury, such as hypoxia or inflammation, resulting in the spilling of the cellular contents into surrounding tissue. A necrotic cell usually undergoes swelling, as it fails to maintain homoeostasis with its environment. However, it is now clear that there is also a genetically regulated necrosis, involving different molecular pathways (necroptosis, parthanatos, ferroptosis, pyroptosis, autolysis, and mitochondrial permeability transition) [1]. Lastly, the term autophagy indicates a process where cellular components are sequestered into lysosomes for degradation before recycling to form new cellular structures or further being processed and used as a source of energy. Autophagy can also result in destruction of the cell, and in this way, it can be referred to as a form of cell death. Neurons can die through a dozen different modes, which include the three mentioned degenerative processes [1].

**Figure 1:**
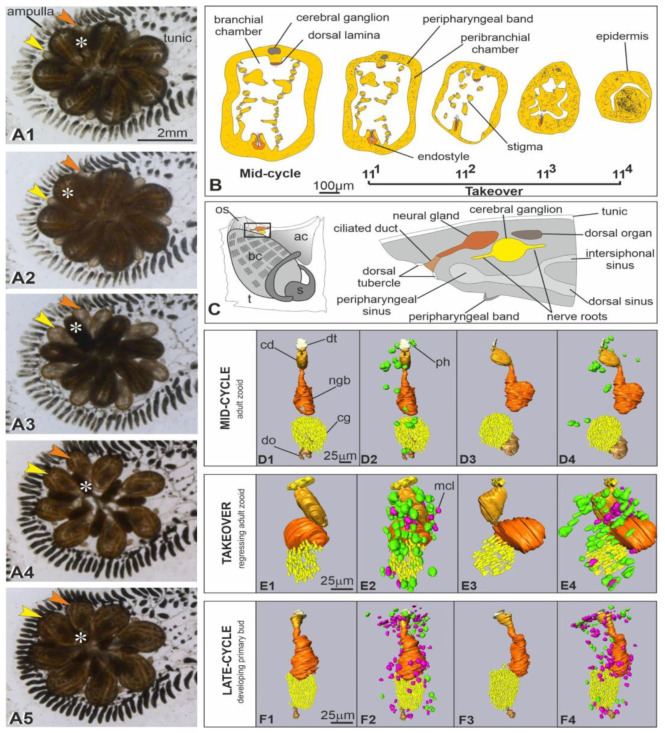
(**A**) Colony of *Botryllus schlosseri* photographed in late-cycle (**A1**) and during takeover. (**A2**): adult zooids in sub-stage 11^1^; (**A3**): sub-stage 11^2^; (**A4**): sub-stage 11^3^; (**A5**): sub-stage 11^4^. The adult zooids (asterisk) progressively reduce to a dark round mass at the system center; in the meantime, primary buds (arrowheads) grow and open their siphons, becoming the new adult generation. Secondary buds are not recognizable In the system, each adult zooid possesses its own oral siphon (not visible in ventral view), whereas the atrial siphon is a common aperture at system center. The yellow and orange arrowheads point at the right and left primary buds belonging to the zooid, marked by asterisk, respectively. Ventral view. a: ampulla; t: tunic. The enlargement is the same in (**A1**–**A5**). (**B**) Illustrations of zooid transverse sections in mid-cycle and during takeover (stages 11^1^–11^4^). In the latter, note the progressive contraction of the adult zooid, the dissolution of inner organs and the increase in immunocytes (black dots). Filter-feeding zooids relay on filtration for feeding and respiration: seawater flows through the oral siphon within the branchial chamber and, passing through the ciliated fissures (stigmata), enters the peribranchial chamber to be expelled (together with fecal pellet) by the atrial siphon. The endostyle produces the mucus entrapping food particles. The peripharyngeal band is a ciliated band at the oral siphon base, separating the branchial chamber with stigmata from the pre-branchial zone, where the dorsal tubercle opens. Scale bar is the same in all illustrations. Modified from [49]. (**C**) Illustration of the neural complex components in a filter feeding zooid. The complex is located at the intersection of the dorsal sinus (which is on the branchial roof, in correspondence of the dorsal lamina), the right and left peripharyngeal sinuses (at the level of the peripharyngeal bands at the oral siphon base), and the intersiphonal sinus (connecting the oral and the atrial siphons) [50]. ac: atrial chamber; bc: branchial chamber; os: oral siphon; t: tunic. (**D**–**F**) 3D reconstructions of the neural complex of a filter-feeding adult individual (mid-cycle, phase 9/8/3; (**D1**–**D4**); 244 sections, 1 μm thick), an adult individual in takeover (phase 11^2^/8/6; (**E1**–**E4**); 74 sections, 1 μm thick), and a late primary bud, just before the takeover (phase 9/8/6; (**F1**–**F4**); 202 sections, 1 μm thick). See Figure S1A for relationships between adult zooid and its bud during the blastogenetic cycle. Ventral (**D1**,**D2**,**E1**,**E2**, anterior at top) and lateral (**D3**,**D4**,**E3**,**E4**, anterior at top, dorsal at left) views. In (**D2**,**D4**,**E2**,**E4**,**F2**,**F4**), immunocytes (green: phagocytes; fuchsia: morula cells) are added with respect to (**D1**,**D3**,**E1**,**E3**,**F1**,**F3**). Organs and cells are color-coded. Scale bar is the same in (**D1**–**D4**,**E1**–**E4**,**F1**–**F4**).

Tunicates manifest apoptosis, necrosis and autophagy throughout their lifespan [9,10,11,12,13,14,15], which includes a mobile larva phase. It exhibits most of the chordate features that tunicates share with vertebrates: a notochord in the tail, a dorsal neural tube giving rise to a tripartite brain [16], a ventral endoderm and a bilateral striated musculature. These traits are lost during metamorphosis, a process during which the tail is completely resorbed through apoptosis [17,18,19,20]. In the colonial tunicate *Botryllus schlosseri*, apoptosis mediates cyclical events of adult resorption, occurring in a phase called takeover [9,10,11,15,21,22,23,24] (Figure 1A,B). In this species, three asexually derived (blastogenetic) generations of zooids (blastozooids) coexist in a colony: the adult, filter-feeding individuals, their buds (primary buds), and a generation of small buds (secondary buds, or budlets) emerging from primary buds (Figure 1A and Figure S1A). Weekly, at 18 °C, a change of generation occurs (takeover phase): the adult individuals die simultaneously and are resorbed by the colony; the primary buds open their siphons, beginning filtration while becoming the new generation of adult zooids; and the secondary buds become primary buds and develop a new generations of budlets. During the takeover, which lasts ~48 h, apoptosis occurs only in the adult generation, and it is functional to bud development [25,26]. This recurrent and massive natural degeneration makes *B. schlosseri* a suitable candidate for studying different pathways of cell death at the organism level on a weekly basis, without the necessity of experimentally inducing it. Conversely, necrosis is the cell death modality occurring in *B. schlosseri* colony senescence [10]. The latter process, involving all of the asexually derived generations of aged colonies, lasts about one week and proceeds according to a series of characteristic changes, such as vascular constriction and congestion, massive pigment cell accumulation in several districts, gradual zooid shrinkage, loss of colony architecture, and ultimately death [10,27,28]. Notable is that although both the takeover and the senescence are processes limited in time, they are both prepared by progressive degenerative events particularly evident in the nervous system [7,29]. In the days preceding takeover and during aging, zooids undergo a decrease in neuron number in their brain, reduced behavioral response to stimuli, and a significant change in the expression of mammalian homologous genes associated with neurodegenerative diseases [7]. However, a detailed analysis at both the ultrastructural and transcriptional level of neurodegeneration during the takeover has not yet been reported. Regarding autophagy, in *B. schlosseri*, the transcript of the vertebrate orthologous gene *Ambra1*, which has a central positive role in regulating autophagy [30,31], has been characterized structurally and phylogenetically [12]. Moreover, the *Ambra1* gene was found expressed at different levels during the asexual phases [12]. At an ultrastructural level, some autophagic figures were recognized during senescence [10]. In vertebrates, *Ambra1* is involved not only in autophagy but also in other key processes such as apoptosis, cell proliferation and nervous system development [32].

In this work, taking advantage of the unique blastogenetic cycle of *B. schlosseri,* as characterized by the recurrent takeover phase and the presence of different cell death mechanisms (apoptosis, necrosis, autophagy) in various developmental phases of its lifespan, we aimed to characterize the morphological, behavioral and transcriptional events of neural degeneration. In this species, the brain, called the cerebral ganglion, is an ovoid structure, located in the dorsal body wall, between the oral and the atrial siphon [7,33,34]. The ganglion is composed of an external cortex containing neuronal somata and an inner medulla of closely packed neurites. It is associated with a sac-like structure, the neural gland, which is believed to be involved in regulating the volume of zooid internal fluid [35,36] and has an active role in adult neurogenesis [7]. A third component, the dorsal organ (whose function is unknown), is associated with the neural gland and the ganglion. Together, these three bodies constitute the neural complex (Figure 1C).

Here, we show that neurodegeneration in *B. schlosseri* occurs involving neuronal cell death forms that are typical in different human neurodegenerative diseases [1]. Neurodegeneration in humans is characterized by the abnormal accumulation of unfolded or misfolded proteins, thus interfering with both neuronal homeostasis and axonal transport [37,38] and driving different types of cellular death [39], along with a cell-to-cell, prion-like transmission of pathological proteins [40,41,42]. Indeed, in *B. schlosseri*, we found that neurodegeneration precociously impairs zooid movement ability and relies on different types of neuronal death, namely apoptosis, regulated necrosis (necroptosis and lysosomal cell death), and autophagy. Phagocytes, involved in neuron clearance, and morula cells, involved in immunotoxicity [43], are recruited and participate in the neurodegenerative process, which exhibits overall features typical of human conformational disorders. Considering these results and the close phylogenetic relationship between tunicates and vertebrates, we propose *B. schlosseri* as a translational model for future studies on neurodegeneration in the organism as a whole and for pharmacological and pre-clinical tests on human neurodegenerative disorders. Moreover, possessing a unique life cycle characterized by the coexistence of neurodegeneration events paralleling brain development, *B. schlosseri* offers the possibility of understanding how cell death is regulated and which crucial homeostatic mechanisms are required to maintain neural tissues, organ size, and function in growing buds.

## 2. Materials and Methods

### 2.1. Mariculture, Sample Collection

Specimens of *Botryllus schlosseri* (family Botryllidae, order Stolidobranchiata) were collected in the Lagoon of Venice (IT) and Monterey Bay (USA). Within a colony, blastozooids are grouped in star-shaped systems, converging toward a center with their cloacal siphons, opening together in a common atrial siphon (Figure 1A). The colonies collected in Venice Lagoon were reared adhering to glass following Sabbadin’s (1955) technique at a constant temperature of 18 °C. The offspring of the colonies collected in Monterey Bay were reared as described in Kowarksy et al. 2021. Thanks to the transparency of colonies, the daily development of buds and zooids was monitored in vivo under a light stereomicroscope in order to select the appropriate sub-stages. Stages are referred to [44].

### 2.2. Behavioral Test

The siphon stimulation test performed, as described in [7], involved the stimulation of the oral siphon epidermal receptors, i.e., primary sensory cells located in the oral siphon wall. Briefly, the test consists of a mechanical stimulation of the outer siphon wall with a solution jet generated by a microinjector. More specifically, we used a glass needle prepared with a Sutter P-87 capillary puller, mounted on a WPI M3301R manual micromanipulator. The same needle was used for all of the stimulations. The water jet used to stimulate the zooids was a solution of 0.5% Phenol red solution in filtered seawater. Each water jet (impulse) was produced in approximately 1 min intervals to allow the zooid to recover and return to a relaxed condition. In this way, each impulse could be considered as “single”, avoiding problems of habituation or sensitization. The jet pressure was gradually increased: starting from a minimum value of 001 kPa, at which no behavioral response was observed, the pressure was increased by 001 kPa each time. Impulses were repeated until the pressure was sufficient to cause oral siphon contraction, at which point the pressure value was recorded. The response to the tests was verified in 20 adult zooids belonging to 4 different colonies in late-cycle and takeover of less than 1-year-old colonies.

### 2.3. Histology and Transmission Electron Microscopy (TEM)

Colonies were fixed for 2 h in 1.5% glutaraldehyde in 0.2 M sodium cacodylate and 1.6% NaCl buffer. After 3 washes in 0.2 M sodium cacodylate and 1.6% NaCl buffer, samples were post-fixed for 1.5 h in 1% OsO_4_ in 0.2 M cacodylate buffer at 4 °C. Colonies were then cut into small pieces, dehydrated and soaked in Epon and propylene solution at 37 °C, 45 °C, and 60 °C. They were then embedded in resin, oriented and sectioned using a Leica ultramicrotome. Serial sections (both cross and sagittal), 1 μm thick, were stained with toluidine blue. For TEM observations, ultra-thin sections (60–80 nm thick) were contrasted with uranyl acetate and lead citrate. Photomicrographs were taken with a Tecnai G^2^ (FEI) transmission electron microscope operating at 100 kV. Images were captured with a Veleta (Olympus Soft Imaging System) digital camera. Some colonies were also fixed for 2 h in Bouin’s solution, rinsed in PBS, dehydrated, and embedded in Paraplast X-TRA (Oxford Labware). Sections (7 μm thick) were cut with a Leitz 1212 microtome, stained with hematoxylin and eosin, and observed under a Leitz Dialux 22 light microscope.

### 2.4. Neural Complex Three-Dimensional Reconstructions

For the study of neural complex component relationships and dynamics throughout the blastogenetic cycle, we produced a new dataset of serial sections for primary buds, whereas we used previously prepared datasets for neural complexes of adult individuals [7]. Briefly, we fixed and embedded in resin a late primary bud (colony phase 9/8/5), just before the takeover phase, and adult individuals in mid-cycle (colony phase 9/8/3) and takeover (the two latter belonging to the same genotype) (Figure S1A). Then, the specimens were serially cut using a Histo Jumbo Knife (Diatome). Sections, 1 μm thick, were stained with toluidine blue, serially photographed by Leica DMR optical microscope, and manually aligned using Adobe Photoshop CS. The Amira software (Thermo Fisher Scientific, Waltham, MA, USA) was used to create 3D reconstructions.

### 2.5. Bioinformatic Analyses

#### 2.5.1. Gene Counts

The zooid gene count data set was produced by RNAseq analysis described in [29]. Gene count determination was performed using a Snakemake pipeline [45] as described in detail in [29]. No brain transcriptomes were produced from regressing zooids, as during the take-over stage, zooid shrinkage compromises the ability to extract the brain. The gene counts were compiled in a tabular format for each of our final comparisons: late-cycle zooids vs. regressing zooids and regressing zooids vs. primary buds at takeover stage.

#### 2.5.2. Gene Ontology

Gene homology was determined based on the genome annotation [6] (http://botryllus.stanford.edu/botryllusgenome/browse/ accessed on 31 January 2023). Briefly, the protein sequences were compared (blastp, evalue < 1 × 10^−10^) to human and mouse proteomes (UniProtKB/Swiss-Prot) and to (blastx, evalue < 1 × 10^−10^) the NCBI non-redundant protein database (nr). For each gene, two annotations were produced: the best hit in nr and the best hit from mouse/human proteome (if present).

#### 2.5.3. Heatmaps

Using GeneAnalytics PathCard [46], we selected genes involved in neuronal cell death and human prion disease (Tables S1 and S2). In order to investigate which genes were differentially expressed during the takeover, the gene count information was imported into R, and homologous genes significantly (*p* < 0.05) upregulated (log2FC > 0) or downregulated (log2FC < 0) were selected. Differential expression between two groups was performed using Deseq2 [47]. Where multiple GIs were associated with the same gene, we preferentially selected GIs based on the longest transcript and completeness data as in Anselmi et al., 2022. The complete list of *B. schlosseri* sequence identifiers (GIs) and associated gene names differentially expressed in the different comparisons can be found in Tables S3–S5.

## 3. Results

### 3.1. Adult Zooid Resorption Is Accompanied by Behavioral Impairment at Takeover

**Figure 2:**
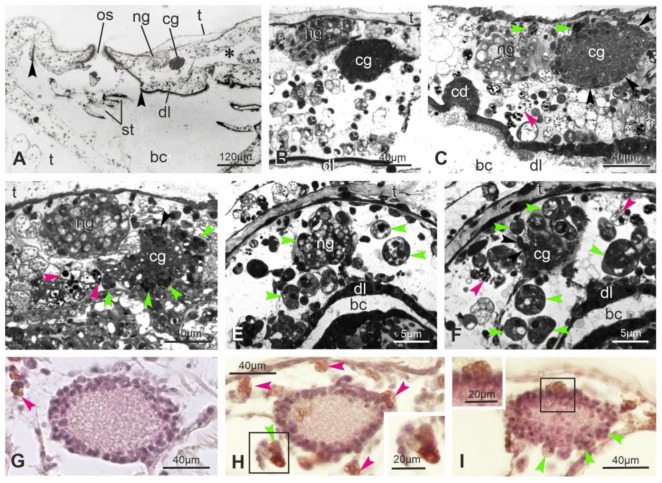
(**A**) Sagittal medial section of an adult filter-feeding zooid (mid-cycle), showing the neural complex located posteriorly to the oral siphon (os). In the neural complex, the neural gland (ng) is dorsal to the cerebral ganglion (cg). The neural gland duct, the dorsal tubercle, and the dorsal organ are not in the section. The asterisk points to the crossroad between the intersiphonal sinus and the dorsal sinus. Anterior at left; dorsal at top. Toluidine blue. arrowheads: peripharyngeal band; bc: branchial chamber; dl: dorsal lamina; st: stigmata; t: tunic. (**B**–**F**) Details of sagittal (**B**–**D**) and cross (**E**,**F**) sections showing the neural complex during takeover, at sub-stages 11^1^ (**B**), 11^2^ (**C**), 11^3^ (**D**) and 11^4^ (**E**,**F**). First signs of epithelial disorganization are recognizable in the dorsal lamina (dl) close to the complex at the beginning of takeover (**B**). The cerebral ganglion (cg) decreases progressively in size and number of neurons (black arrowheads). The neural gland (ng) maintains its vacuolated structures until advanced takeover. Note the increasing number of immunocytes, phagocytes (green arrowheads) and morula cells (pink arrowheads), close to the degenerating neural complex. Toluidine blue. bc: branchial chamber; cd: ciliated duct of the neural gland; dl: dorsal lamina; t: tunic. (**G**,**H**) Cross paraffin sections of the cerebral ganglion in adult filter-feeding zooids (mid-cycle) (**G**), and at sub-stages 11^1^ (**H**) and 11^2^ (**I**) to show the increase in number of immunocytes, phagocytes (green arrowheads, inset in **H**) and morula cells (pink arrowheads, inset in **I**), close to it and infiltrated among neurons. Hematoxylin and eosin.

The takeover is the resorption phase, which involves all of the tissues of adult zooids, including the neural complex (Figure 1, Figure 2 and Figure S1). The regressing zooid stage (Stage 11; [48]) was subdivided into four sub-stages, based on the main anatomical events characterizing the colony (Table 1).

At the end of the takeover, the zooids are spherical dark masses that will be resorbed in a few hours [11]. In the meantime, primary buds grow to adulthood, opening their siphons and beginning filtration to sustain the colony (Figure 1A,B). During takeover, the hemolymph circulation is guaranteed by beating hearts of both primary buds and regressing adult zooids; however, at the end of the takeover, the circulation depends solely on the newly formed bud hearts.

Relying on methods performed in earlier works [7,51], we stimulated the oral siphon epidermis mechanoreceptors to analyze zooid behavioral performances. These sensory cells have both an apical cilium (dendrite) embedded into the tunic and an axon directed to the brain. When stimulated, they activate the oral siphon sphincter muscle contraction and induce the oral siphon closure. Using a waterjet controlled by a microinjector, we stimulated these mechanoreceptors on filter-feeding zooids and at the beginning of takeover (sub-stage 11^1^) to determine the minimum pressure needed to induce the oral siphon contraction, as a quantitative parameter to measure mechanoreceptor sensitivity. If the minimum pressure needed to provoke the siphon contraction in a filter-feeding zooid is on average 3.125 psi (21.546 kPa), we found that once the oral siphon closes, the zooid does not respond to the mechanical stimuli, and no movement is produced.

### 3.2. At Takeover, the Neural Complex Progressively Is Infiltrated by Immunocytes, Reduces in Size and Loses Its Organization

The neural complex (neural gland, cerebral ganglion and dorsal organ) in filter-feeding zooids is located in the dorsal body wall, between the oral and atrial siphons, in a well vascularized area (Figure 1C and Figure 2A). Indeed, the complex lies in the connective tissue at the intersection of three sinuses guaranteeing hemolymph circulation in the oral siphon, dorsal body wall, and dorsal branchial chamber [50] (Figure 1C and Figure S1). Some immunocytes (phagocytes and some morula cells) can be recognized in the hemolymph lacuna surrounding but rarely in contact with the complex [7] (Figure 1D, Figure 2G and Figure S1B).

The ganglion is about 80 μm long, the neural gland is about 140 μm, and the dorsal organ is 75 μm long (Figure 1D and Figure 2A,G). The neural gland exhibits a spongy sac-like body opening anteriorly into the prebranchial region (the area located between the oral siphon and the first row of stigmata in the branchial chamber) through a ciliated duct that enlarges as a funnel and protrudes into the region as dorsal tubercle. Different from the dorsal lamina cilia, which point toward the chamber bottom, those of the duct bend posteriorly, toward the neural gland body. The latter has vacuolated cells, such that its lumen is not easily recognizable. Where the gland is in contact with the cerebral ganglion (which is ventral to the gland), gland cells have an epithelial aspect and do not show vacuoles, appearing like close neurons, such that the exact border between the gland and the ganglion is not recognizable. The posterior-most part of the gland has an epithelial aspect and elongates in a short cord toward the dorsal organ. The latter is an ovoidal structure with a small lumen. The organ is in some points in continuity with the ganglion, and cells belonging to the two structures show no histological differences. The dorsal organ is a dynamic structure, and in the late-cycle, it is no longer recognizable in most of the zooids. The cerebral ganglion is ovoid, with a regular profile defined by the arrangement in 2–3 layers of the neuronal somata (forming the ganglion cortex), closely in contact with each other and in some points also with the neural gland and the dorsal organ. Neurites form a densely packed medulla. At the beginning of the takeover (sub-stage 11^1^; Figure 1A,B, Figure 2B and Figure S2), the neural complex still maintains its anatomical integrity, although the ganglion displays a lower number of neurons, and some immunocytes are now in the surrounding hemocele (Figure 2H). Moreover, some alterations can be seen in the adjacent zooid organs; for example, the dorsal lamina (the ciliated band on the roof of the branchial chamber involved in the formation of the rich-in-particle mucus cord moved toward the gut) epithelium exhibits cells of different affinity for the histological labeling, indicating that cytoplasmic alterations are occurring (Figure 2C and Figure S2).

In the following takeover hours (sub-stage 11^2^; Figure 1A,B,E and Figure 2C), there are still no significant changes in the neural complex histology. Nonetheless, the zooid contraction dislocates the complex components, such that the body gland can be found anterior to its duct (Figure 1E and Figure S1C). Indeed, the branchial chamber is smaller than in the previous sub-stage, and ciliated epithelia (e.g., dorsal lamina and stigmata) show irregular ciliary arrangements. Moreover, immunocytes close to the complex are more numerous than previously (Figure 1D–E, Figure 2I, Figures S1C and S3).

As regression proceeds (sub-stage 11^3^; Figure 1A,B, Figure 2D and Figure S3), the neural gland still maintains its histological structure, characterized by highly vacuolated cells; however, the cerebral ganglion exhibits a remarkably lower number of neurons. The pharynx is completely infiltrated by phagocytes, and the branchial epithelium profile is no longer recognizable, together with the cilia that previously characterized the branchial stigmata and the dorsal lamina. Nonetheless, cilia belonging to the neural gland ciliated duct are still recognizable. In following hours, as the zooid contraction proceeds, the stigmata are no longer recognizable in the branchial chamber, whose lumen is rich in phagocytes (Figure S4). Still, the ciliated duct bears cilia, and the neural gland maintains its histological integrity. However, in the ganglion, neuronal somata are very few and no longer packed, and the medulla is no longer compact, showing vacuolation (Figure S4I). Some phagocytes infiltrate the ganglion. In general, there is a significant imbalance between the body size and the neural complex size: the latter is still recognizable in its components, whereas most of the other zooid organs are no longer present.

Usually, when the regressing zooids are at about this takeover sub-stage, their primary buds have completed their growth. All of the inner organs, including the neural complex (Figure 1F and Figure S5), are mature for the physiological activities characterizing adult life, such as filter-feeding and respiration. The siphon opening determines the passage of the primary bud to the adult zooid stage.

In the terminal regression (sub-stage 11^4^; Figure 1A,B, Figure 2E,F and Figure S6), when the hearts no longer beat in regressing zooids, these are dark pigmented spherical structures. A residual lumen marks the branchial chamber where the ciliated duct still opens. The neural gland is very small and difficult to identify; several phagocytes are close to it. The ganglion is also very small, and a few neurons surrounding a residual medulla are recognizable in sections. Although all other zooid tissues are no longer recognizable, both the heart and the neural complex components remain until the end of the degeneration process.

### 3.3. Apoptosis, Regulated Necrosis, and Autophagy Characterize the Neural Complex Degeneration

**Figure 3:**
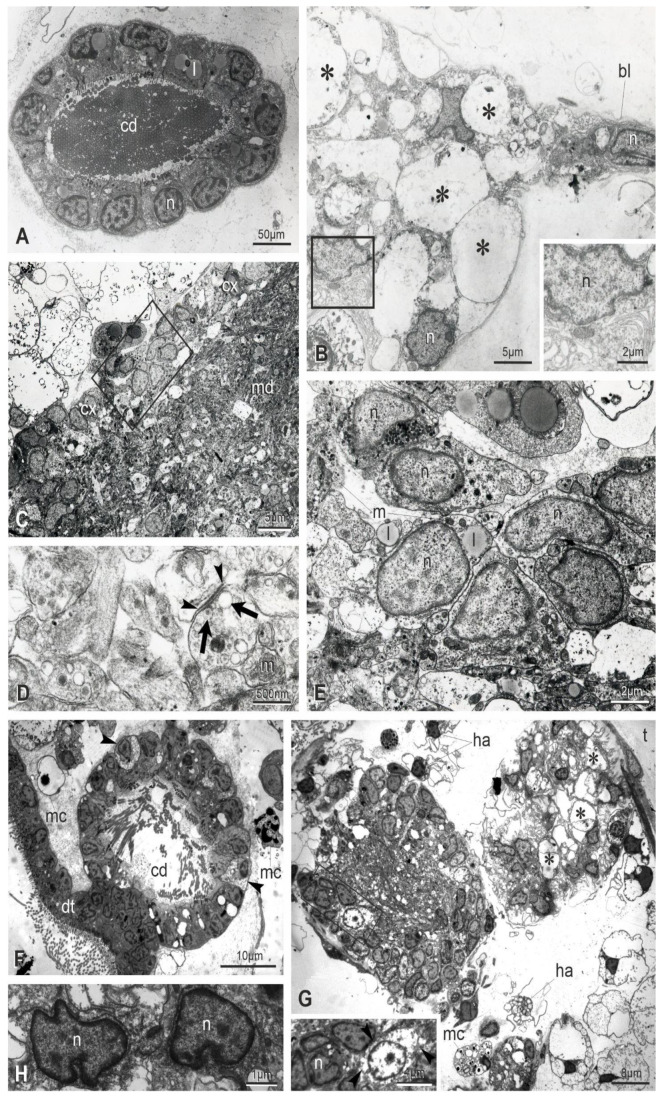
Ultrastructure of the neural complex in filter-feeding zooid (mid-cycle) (**A**–**E**) and in a regressing zooid at sub-stage 11^1^ (**F**–**H**). TEM. (**A**) Thin sections revealing that the ciliated duct (cd) of the neural gland is highly ciliated. l: lipid droplet; n: nucleus in a ciliated duct cell. (**B**) Section through the main body of the neural gland showing its posterior-most part, elongating in an epithelial-like short cord (right). Cells are provided with basal lamina (bl). In the gland body, cells have basally oriented nuclei (n) (enlarged in inset) and large vacuoles (asterisks). (**C**–**E**) Cerebral ganglion. A cortex (cx) of 2–3 layers of neuron somata surrounds a medulla (md) of packed neurites. Squared area in (**C**) is enlarged in (**E**) to show some neurons with nuclei (n) with regular profile and granules and mitochondria (m) in their cytoplasm. In the medulla, synaptic contacts are observed with characteristic presynaptic vesicles (arrows) and postsynaptic densities (arrowheads). l: lipid droplet. (**F**) The ciliated duct (cd) exhibits some swollen cells (less electron-dense than others, arrows) and lobed nuclei with condensed chromatin close to the nuclear membrane. dt: dorsal tubercle; mc: morula cell. (**G**) Cells of both the cerebral ganglion (left, inset) and the neural gland (right) show ultrastructural changes at nucleus level, such as irregular profile and chromatin condensed close to the nuclear membrane. Inset: neuron with a lobed nucleus (n) close to a necrotic neuron (arrowheads) displaying cytoplasm with vacant spaces and nucleus with rarefied chromatin. Asterisks: vacuoles in neural gland cells; t: tunic. (**H**) Detail of nuclei (n) in two neural gland cells. ha: hyaline amebocyte, a phagocyte precursor; mc: morula cell.

The ultrastructure of the neural complex in filter-feeding zooids has been previously described [33]. Briefly, the neural gland ciliated duct has cuboidal cells, arranged in a single layer and equipped with basal round nuclei and apical microvilli and cilia (Figure 3A). Numerous mitochondria, some lipid drops and glycogen granules are in the cytoplasm. The ciliated, funnel-like duct narrows in a non-ciliated part connecting to the neural gland body. The latter has highly vacuolated cells, with round nuclei, Golgi fields and endoplasmic reticulum cisterns confined at the cell periphery (Figure 3B). The dorsal organ has cuboidal cells, similar in aspect to the epithelial cells of the posterior neural gland. The cerebral ganglion is bordered by a fibrous sheet, interrupted only where the ganglion is in continuity with the adjacent neural gland and dorsal organ. In the cortex, neurons possess a large round/ovoidal nucleus, numerous mitochondria and some lipid droplets (Figure 3C). In the medulla, neurites are strongly intertwined and characterized by microtubules and some mitochondria. Neurotransmitter vesicles are concentrated mostly in synaptic areas, recognizable by the narrow cleft between the presynaptic and postsynaptic membranes (Figure 3D). The postsynaptic membrane is thicker than the presynaptic one; the latter is associated with neurotransmitter vesicles.

The ultrastructural observations allow one to recognize signs of degeneration at the subcellular scale even in early takeover (sub-stage 11^1^), not appreciable in histological sections. These signs affect zooid epithelia, such as the epidermis, the peripharyngeal band, and the dorsal lamina (Figure S7). In the latter, some cells are swollen, exhibiting loss of ribosomes and vacant spaces in the cytoplasm. Furthermore, cells have nuclei with condensed chromatin close to the nuclear membrane; their shape is in some cases lobed. Similar alterations are displayed by ciliated duct cells (Figure 3F). With respect to the filter-feeding zooids, the neural gland body maintains its vacuolated structure (Figure 3G,H and Figure S7); however, some nuclei have an irregular shape and chromatin condensed at the periphery. A comparable situation is exhibited by some neurons in the cerebral ganglion, indicating that apoptosis, necrosis and autophagy are affecting them. Some dark degraded autophagic vacuoles can be recognized (Figure S7C); moreover, some neurons are swollen, exhibiting loss of ribosomes and vacant spaces in the cytoplasm, whereas others show lobed nuclei with condensed chromatin (Figure 3G and Figure S7C). In the medulla, typical synapses can be recognized, but neurites are filled with small vesicles, ranging from 25 nm to 100 nm in diameter, and autophagic figures can be seen (Figure S7I).

**Figure 4:**
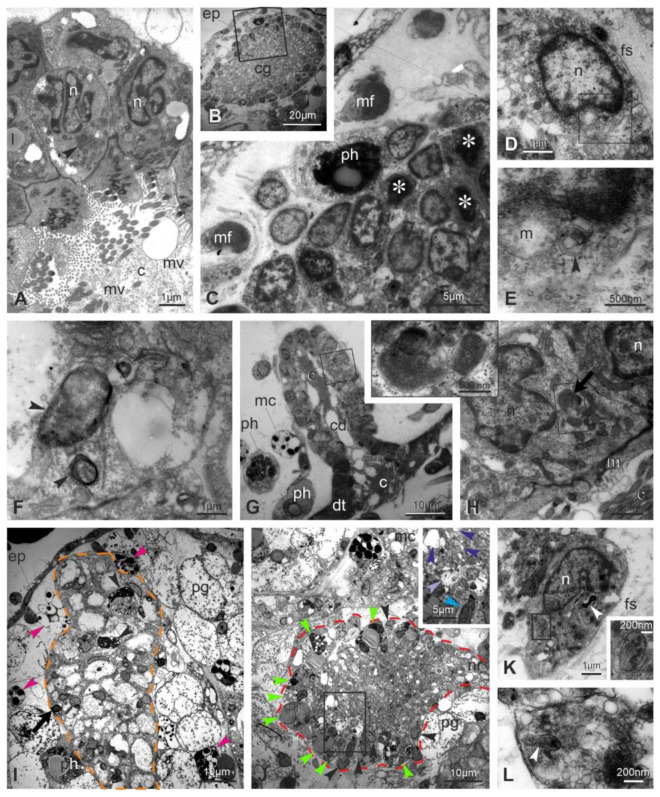
Ultrastructure of the neural complex in regressing zooids at sub-stages 11^2−3^ (**A**–**F**) and 11^3^ (**G**–**L**). TEM. (**A**) Ciliated duct cells show lobed nuclei (n). At their apex, both microvilli (mv) and cilia (c) project into the lumen duct. Arrowhead: glycogen granules; l: lipid droplet. (**B**,**C**) Cerebral ganglion (cg) displaying numerous pyknotic nuclei (asterisks). Squared area in (**B**) is enlarged in (**C**). ep: epidermis; mf: muscle fiber; ph: phagosome in a partially cut phagocyte. (**D**,**E**) Neuron exhibiting an autophagosome (arrowhead) close to its nucleus (n). Squared area in (**D**) is enlarged in (**E**). fs: cerebral ganglion fibrous sheet; m: mitochondrion with swollen cristae. (**F**) Autolysosomes (arrowheads) with single membrane and electron-dense degraded content are in a neural gland cell. (**G**,**H**) Ciliated duct (cd) degeneration: note in (**G**) the number of lobed and pyknotic nuclei and the agglutinated cilia within the duct lumen. Squared area in (**G**) is enlarged in (**H**) to show a ciliated duct cell exhibiting an autophagosome (arrowhead) close to an autolysosome with single membrane and electron-dense degraded content (arrow). Both of the autophagic figures are enlarged in inset; note the autophagosome double membrane. c: cilia; dt: dorsal tubercle; m: mitochondria; mc: morula cell; nu: nucleus; ph: phagocyte. (**I**) Degenerating neural gland (orange dotted line) with pyknotic nuclei (black arrow) and autophagic figures (black arrowheads). The gland is surrounded by pigmented cells (pg) and morula cells (pink arrowheads). ep: epidermis; ph: phagocyte. (**J**) Cerebral ganglion (red dotted line) whose cortex presents several phagocytes (green arrowheads) among few neurons (black arrowheads). The medulla is vacuolated. Inset: neuron with lobed nucleus (light blue arrowhead), swollen neuron (violet arrowhead), and medulla vacuoles (dark blue arrowheads). The surrounding hemocele is filled with pigmented cells (pg) and cell debris. mc: morula cell; nr: nerve root. (**K**) Neuron with lobed nucleus (n), a degradative multilamellar body (white arrowhead) and an autophagosome (enlarged in inset to show its double membrane). fs: cerebral ganglion fibrous sheet. (**L**) Neurite filled with vesicles of different sizes. Arrowhead: tubular structure.

As the takeover progresses (sub-stage 11^2−3^), signs of alteration increase, confirming that multiple modes of cell death are involving the neural complex and adjacent tissue (Figure 4). Both the ciliated duct cells and the ganglionic neurons exhibit nuclei more polymorphic and pyknotic than previously, indicating apoptosis (Figure 4A–E). Moreover, in the ganglion neurons, swollen neurons with vacant cytoplasm are present as evidence of necrosis (Figure 4J). Autophagic figures, such as autophagosomes delimited by a double membrane (Figure 4E,K), autolysosomes with single membrane and electron-dense degraded content (Figure 4F,H,I), and electron-dense degradative multilamellar bodies (Figure 4K), characterize cell cytoplasm of the neural complex components. Moreover, some mitochondria show swollen cristae (Figure 4E). The medulla is vacuolated, and neurites are filled with vesicles (rarely seen elongated as tubules) of different size, not associated with synapses (Figure 4J,L). The neural complex is surrounded by numerous hemocytes, including large phagocytes, morula cells and pigmented cells, the latter being responsible for the dark pigmentation of the regressing zooid (Figure 4I,J). In particular, the ganglion, which contains a few neurons, is infiltrated by phagocytes containing large phagocytic vacuoles with cell debris (Figure 4J).

**Figure 5:**
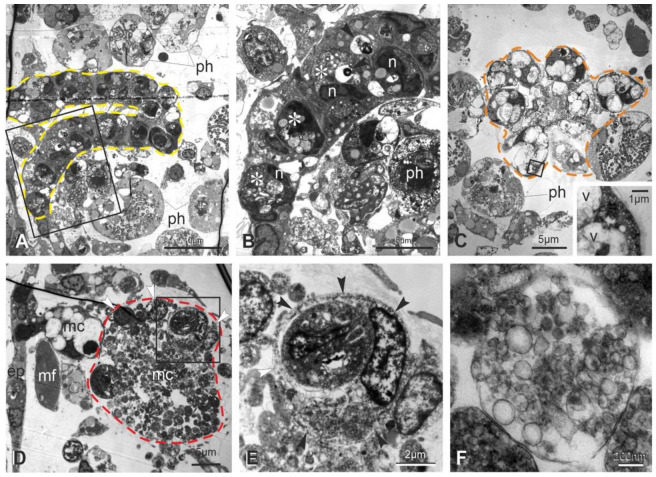
Ultrastructure of the neural complex in regressing zooids at sub-stages 11^4^. TEM. (**A**,**B**) Ciliated duct (yellow dotted line in **A**); squared area in (**A**) is enlarged in (**B**). Severe signs of cell degeneration are represented by lobed and pyknotic nuclei (n), large autolysosomes (asterisks), degradative multilamellar bodies (arrowheads). Phagocytes (ph) are in the surrounding hemocele. (**C**) Neural gland constituted of very few cells. Squared area is enlarged in inset to show in cytoplasm both small vesicles and degradative vacuoles among large vacuoles (v). Phagocytes (ph) are in the surrounding hemocele. (**D**–**F**) Cerebral ganglion (red dotted line in **D**) containing very few degenerated neurons (white arrowheads). The medulla (md) is loose and vacuolated, and neuritis (**F**) are filled with small vesicles ranging between 25 and 100 nm in diameter. Squared area in (**D**) is enlarged in (**E**) to show a phagocyte (black arrowheads) infiltrated into the ganglion. ep: epidermis; mc: morula cell; mf: muscle fiber.

At the end of the takeover (sub-stage 11^4^), the ciliated duct cells display highly pyknotic and lobed nuclei and large autophagic figures in the cytoplasm; cell organelles are no longer recognizable (Figure 5A,B). The neural gland body is extremely reduced and composed of very few vacuolated cells; among vacuoles, the residual cytoplasm contains small vesicles, but organelles (such as ribosomes or reticulum cisterns) are not recognizable (Figure 5C). Very few neuronal somata are in the cerebral ganglion (Figure 5D,E), and its vacuolated medulla is reduced to a few neurites filled with small vesicles. Numerous phagocytes surround and infiltrate the neural complex cell components.

### 3.4. Transcriptome Analyses Suggest That Apoptosis, Necroptosis, Lysosomal Cell Death, and Autophagy Are Involved in Neurodegeneration

The ultrastructural identification of neuronal degeneration features associated with different types of neuronal cell death prompted us to search for molecular signatures potentially linked to these events in previously produced RNAseq datasets of brain, zooids and buds samples [7,29,52] by conducting new comparisons and analyses.

**Figure 6:**
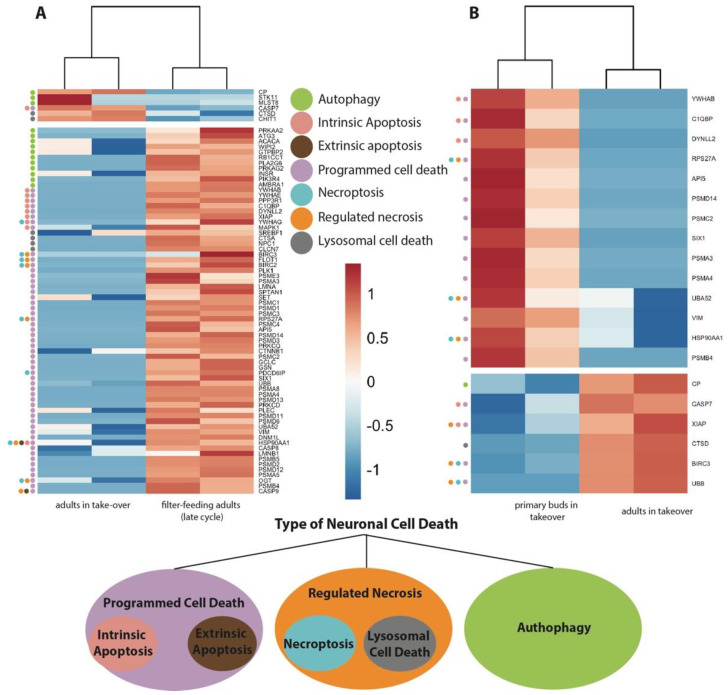
Putative homologous genes associated with neuronal cell death are differentially expressed in zooids during the weekly cycle when comparing filter-feeding adults (late-cycle) and adults in takeover (**A**) and comparing adults in takeover with primary buds in takeover (**B**). Colored dots on the heatmap left indicate the cell death forms in which the genes are involved. Color scale in A and B depicts log2CPM normalized expression values scaled by row.

We analyzed the transcriptomes of: adult filter-feeding zooids (late-cycle; *n* = 2), adult zooids at takeover (*n* = 2), and primary buds at takeover (*n* = 2) (Figure 6 and Table S1). We compared the transcriptomes of (1) adult zooids at takeover vs. filter-feeding zooids (Figure 6A), and (2) adult zooids at takeover vs. their primary buds (Figure 6B). The first comparison aimed to identify genes responsible for adult zooid neurodegeneration, while the second investigated transcriptional differences between individuals coexisting at takeover, but subject to a different fate (regression in the adults vs. development in the buds). We investigated differentially expressed genes related to apoptosis (both intrinsic and extrinsic), necroptosis, lysosomal cell death, and autophagy. Comparing adults in takeover vs. filter-feeding zooids, we identified 74 genes differentially expressed, while when comparing the adults in takeover vs. the buds, we identified 20 genes differentially expressed.

Apoptosis is triggered by two principal pathways: the intrinsic (or mitochondrial) pathway and the extrinsic (or death receptor) pathway. The extrinsic pathway begins outside a cell, when conditions in the extracellular environment determine that a cell must die. In contrast, the intrinsic pathway begins when an injury occurs within the cell and the resulting stress activates the apoptotic pathway. We found more differentially expressed genes belonging to the intrinsic pathway (nine genes) vs. two belonging to the extrinsic pathway. The latter are genes also shared by the necrosis pathway. Within the genes differentially expressed in the programmed cell death pathways and more highly expressed in filter-feeding zooids than takeover, we found genes involved in apoptosis inhibition, pro-apoptosis and apoptosis regulation (Figure 6A). Those that are involved in apoptosis inhibition include apoptosis inhibitor 5 (*API5*) [53], polo-like kinase 1 (*PLK1)* [54], γ-glutamyl-cysteine ligase catalytic subunit (*GCLC*) [55], homeodomain protein (SIX1), Baculoviral IAP Repeat Containing 3 (*BIRC3* and *BIRC6*) [56], X-Linked Inhibitor of Apoptosis (*XIAP*) [57], and Tyrosine 3-Monooxygenase/Tryptophan 5-Monooxygenase Activation Protein Beta (*YWHAB*) [58]. Among the genes involved in regulation of apoptosis, we found template activating factor 1 (*SET)*, O-N-acetylglucosamine transferase (*OGT*), Mitogen-Activated Protein Kinase 1 (*MAPK1*), and Tyrosine 3-Monooxygenase/Tryptophan 5-Monooxygenase Activation Protein Gamma (*YWHAG*). Within the genes with lower expression in filter-feeding zooids than in adults in takeover, we found *CASP7*, a pro-apoptosis gene involved in cell membrane proteins, nucleus and cytoplasm cleavage [59]. Among the genes differentially expressed in *B. schlosseri*, comparing buds in takeover with adults in takeover, we found *API5* (Apoptosis inhibitor); *SIX1* a transcription factor that is involved in the regulation of cell proliferation, apoptosis and embryonic development; and *VIM* (Vimentin) whose encoded protein is responsible for maintaining cell shape and cytoplasm integrity and stabilizing cytoskeletal interactions [60]. They are more highly expressed in the bud in takeover as compared to adults in takeover. Conversely, *XIAP* is more highly expressed in adults in takeover as compared to buds in takeover (Figure 6B).

Necrosis has become synonymous with rupture of the plasma membrane, and it has become clear that it can be caused by many different mechanisms. Regulated (or programmed) necrosis has been distinguished from unregulated necrosis in that regulated necrosis is genetically controlled and involves active cellular processes that can (in principle) be blocked, whereas unregulated necrosis involves passive processes (for example, tissue trauma or toxins acting directly on the plasma membrane) that may be difficult or impossible to block. The best-characterized form of regulated necrosis is necroptosis. Necroptosis is defined as a necrotic cell death dependent on the kinase activity of Receptor Interacting Kinase 1 (RIP1), kinase activity of RIP3, and expression of the pseudokinase Mixed Lineage Kinase Domain-like (MLKL). Among the genes differentially expressed in *B. schlosseri* when comparing filter-feeding adults with adults in takeover, greater expression in the former was observed for: (1) YWHAG; (2) BIRC2-3, an IAP family of proteins that inhibit apoptosis; (3) FLOT1 (Flotillin 1), a gene that encodes a protein localized to the caveolae, small domains on the inner cell membranes, and plays a role in vesicle trafficking and cell morphology; and (4) PDCD6IP (Programmed Cell Death 6 Interacting Protein), the overexpression of which, as studies using mouse cells have shown, can block apoptosis. Overexpression of this gene product and endophilins results in cytoplasmic vacuolization, which may be partly responsible for the protection against cell death. Among the genes differentially expressed in *B. schlosseri* when comparing the buds in takeover with adults in takeover, we found: UBB (Ubiquitin B), which has a major role in targeting cellular proteins for degradation by the 26S proteosome, and BIRK 3 with higher expression in adults in takeover; and HSP90AA1 (Heat Shock Protein) and UBA52 (Ubiquitin A-52) with higher expression in the buds in takeover.

Lysosomal cell death (LCD) (also known as autolysis) is defined as cell death resulting from lysosomal membrane permeabilization. LCD is executed mainly by proteases released from lysosomes into the cytosol, particularly including cathepsins B, D, and L, but also other hydrolases. Among the genes differentially expressed in *B. schlosseri* when comparing filter-feeding adults with adults in takeover, we found that: (1) CTSD (Cathepsins D) is differentially more highly expressed in takeover zooids; (2) CHIT1 (Chitinase 1) is secreted by activated human macrophages and is markedly elevated in plasma of Gaucher disease patients. It is differentially more highly expressed in takeover zooids; (3) CTSA (Cathepsins A) are a group of lysosomal proteases that have a key role in cellular protein turnover. They are more highly expressed in filter-feeding zooids than in adults in takeover; (4) NPC1 encodes a large protein that resides in the limiting membrane of endosomes and lysosomes and mediates intracellular cholesterol trafficking. It is more highly expressed in filter-feeding zooids than in adults in takeover; (5) CLCN7, which encodes for a voltage-gated channel mediating the exchange of chloride ions against protons, functions as antiporter and contributes to the acidification of the lysosome lumen and may be involved in maintaining lysosomal pH. It is more highly expressed in filter-feeding zooids than in adults in takeover. Among the genes differentially expressed in *B. schlosseri* when comparing the buds in takeover with adults in takeover, we found only one gene, CTSD, that is more highly expressed in adults in takeover.

Autophagy normally functions to prevent cell death but, if excessive, may cause cell death. Autophagy is a process of a cell “self-eating”, whereby cell constituents are delivered to the lysosome for digestion and recycling. The best-studied type of autophagy is macroautophagy, where delivery of cell constituents to lysosomes occurs via vesicles known as autophagosomes. Autophagosome formation is initiated through a cascade of signals targeting three distinct multi-protein complexes comprising autophagy-related (ATG) genes and additional proteins that are not homologs of the yeast ATG repertoire but are found in multicellular organisms. Among the genes differentially expressed in *B. schlosseri* when comparing the filter-feeding adults with adults in takeover, we found the following more highly expressed in the former: (1) PRKAA1 (protein kinase); (2) ATG3 (autophagy related), which encodes a ubiquitin-like-conjugating enzyme and is a component of ubiquitination-like systems involved in autophagy, the process of degradation, turnover and recycling of cytoplasmic constituents in eukaryotic cells. This protein is known to play a role in regulation of autophagy during cell death; (3) INSR (insulin receptor); (4) AMBRA1, involved in macroautophagy, positive regulation of phosphatidylinositol 3-kinase activity, and response to mitochondrial depolarisation. Among the genes differentially expressed in *B. schlosseri* when comparing the buds in takeover with adults in takeover, we found only one gene: CP (ceruloplasmin), a gene involved in the peroxidation of Fe (II) transferrin to Fe (III) transferrin. It is more highly expressed in adults during takeover.

### 3.5. Genes Associated with Conformational Disorders Are Differentially Expressed during Neurodegeneration

**Figure 7:**
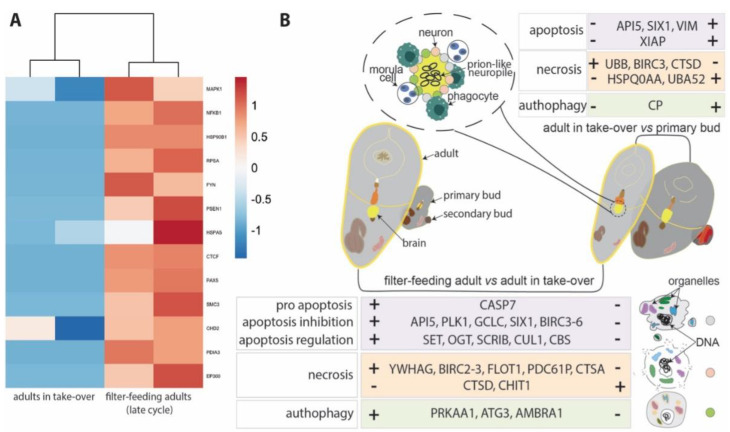
(**A**) Differentially expressed genes associated with prion diseases. Categories are based on the link that each gene has with a neuronal cell death type. The color scale depicts log2CPM normalized expression values scaled by row. (**B**) Key genes involved in the apoptosis, necrosis and autophagy pathways differentially expressed between the filter-feeding adults and adults in takeover (lower box) and between the adults in takeover and the buds (upper box). The illustration on the top shows a brain during takeover composed of a few neurons (gray) and surrounded by phagocytes (green) and morula cells (pink). On the bottom right, the 3 illustrations graphically summarize the 3 different kinds of neuron death.

Our previous data [7] showed that, when approaching takeover and during aging, the neural complex of *B. schlosseri* differentially expresses genes associated with human neurodegenerative diseases, such as Alzheimer’s disease, Parkinson’s disease, frontotemporal dementia, and Huntington’s disease, which are “proteinopathies” exhibiting a prion-like propagation [61,62,63]. In the light of the morphological and transcriptomic data reported above, we decided to investigate if zooids in takeover differentially express genes associated with prion diseases. Prion diseases are characterized by tubulovesicular structures in neurites in the form of vesicles of different size [64,65] and vacuoles in neuropile (responsible for the so-called spongiform aspect of neuropile) [66], both elements that we recognized in the degenerating brain of *B. schlosseri*. We analyzed in GeneAnalytics [46] the homologous genes involved in prion disease pathways that were significantly (*p* < 0.05) upregulated or downregulated when comparing the transcriptomes of adult zooids at takeover (*n* = 2) with filter-feeding adult zooids (*n* = 2) (Figure 7A and Table S1). We found 13 upregulated genes in the filter-feeding adult zooids. Among them, we found PSEN1 (presenilin-1), a key gene in Alzheimer’s disease that regulates APP processing through its effects on gamma-secretase, an enzyme that cleaves APP, producing the protein aggregation; FYN (FYN Proto-Oncogene), a gene that controls cell growth; SMC3 (Structural Maintenance Of Chromosomes-3), a gene that enables proper chromosome segregation; CHD2 (Chromodomain Helicase DNA Binding Protein-2), a gene that alter gene expression possibly by modification of chromatin structure; and EP300 (E1A Binding Protein P300), a gene that regulates transcription via chromatin remodeling and is important in the processes of cell proliferation and differentiation.

## 4. Discussion

As neuronal cell death is a process of critical importance both in nervous system development and in the pathogenesis of neurodegenerative disease, understanding which homeostatic mechanisms regulate it is of crucial importance. Since the classification of cell death [67] into three main categories—apoptosis, necrosis, and autophagy—50 years ago, new forms of neuronal cell death (such as necroptosis, phagoptosis, ferroptosis, and pyroptosis) have recently been described [8]. A new paradigm has emerged over the years [1]: modes of neuron death are numerous and can overlap in their biochemical pathways, making their recognition ambiguous. Environmental factors, such as interactions with neighboring cells, can initiate cell death. Therefore, it is not a “cell autonomous” event; a specific physiological stimulus can trigger multiple cell death forms; the same pathology, acute as with stroke or chronic as with Parkinson’s disease, can concurrently involve different cell death mechanisms. In addition to in vitro approaches, this complexity requires adequate in vivo models.

In this and previous studies [7,68], we showed that *B. schlosseri* is a valuable in vivo model for the study of neurodegeneration in mammals. It is phylogenetically close to vertebrates; easy to rear in a laboratory; characterized by a cycle of short-term, recurring physiological neurodegeneration distinguished by multiple neuronal cell death modes; suitable for testing drugs and chemicals whose effect can be monitored due to the transparency of its tunic and the easily identifiable circulatory system; and marked by a panel of behavioral, morphological and molecular responses correlated with aging. Indeed, young and old colonies of *B. schlosseri* differentially express genes associated with human neurodegenerative diseases, such as Alzheimer’s, Parkinson’s, Huntington, and frontotemporal dementia, showing that a combination of brain degenerative and developmental events occur simultaneously in a number of individuals.

In *B. schlosseri*, apoptosis was previously believed to be the cell death pathway occurring at takeover, which is a genetically regulated event of colony life [9,10,11,15,21,22,23,24], with necrosis and autophagy recognized in colony senescence [10,27,28]. Here, we show that the neural complex and the surrounding tissues, such as the dorsal lamina and the epidermis, exhibit all three modes of cell death (apoptosis, necrosis, and autophagy). We suggest that this first ultrastructural and transcriptomic study, focused on the nervous system and neurodegeneration of *B. schlosseri* at takeover, opens the door to future translational studies on human neurodegenerative diseases.

### 4.1. Cell Death in B. schlosseri: A Cyclical Natural Event for Colony Survival

The regression and resorption of adult zooids in the colony of *B. schlosseri* is a natural event characterizing the blastogenic cycles. Indeed, zooid death is an obligatory phase for the progression of bud development; buds do not grow properly when they are deprived of resources coming from parent resorption [69]. The common circulatory system ensures that buds are provided these necessary resources. Moreover, the takeover prompts a rejuvenation of the whole colony. Although in *B. schlosseri* the tunic and its circulatory system have a high regenerative capability [70], zooids do not regenerate when damaged but are resorbed, a situation distinct from solitary ascidians that are also able to regenerate the brain when ablated [71]. Of particular salience in a colonial tunicate such as *B. schlosseri* is the contemporary presence of developing (bud) and degenerating (adult) zooids in the colony at takeover. Buds rely on adults for their development but are unaffected by their degeneration, although connected to them via a circulatory system that synchronizes both events in the nervous system where the adult brain is regressing and the bud brain is refining its structure, as evidenced by the massive reduction in bud nerve number occurring just before the bud siphon aperture [29,72]. Therefore, we deduced that buds possess defense mechanisms protecting them from the adjacent degeneration. Additionally worth noting is that, in *B. schlosseri*, apoptosis plays a role not only in takeover, but also in the allogeneic resorption [73,74,75].

The takeover has been divided into four steps (sub-stages 11^1^–11^4^, according to Sabbadin, 1955) based on zooid contraction level. In contrast from a previous report [11], in our histological analyses, we found that the degeneration process is already present as soon as the takeover begins. Our results suggest that there is a continuum of progressive degenerative modifications that start in the filter-feeding zooid in late-cycle [7]. In the same colony, individuals are often not perfectly synchronized in regression; some may show more advanced alterations than others, even if individuals are clones. We confirmed that the takeover involves an initial event of zooid contraction, drastically reducing the branchial and peribranchial chamber volume [49]. With respect to cell degeneration, an anterior to posterior gradient was previously proposed, affecting firstly the branchial chamber, then the stomach and the intestine [9,49]. In the neural complex (which is an anterior structure), the first signs of cell alteration appear in early takeover; however, the complex remains recognizable until the takeover ends, when other organs are no longer present. In the present analysis, the neural complex recognition was possible only thanks to the analyses of histological serial sections. At the end of takeover, the complex is very small, and there are no anatomical references that can be used to orient the section analyses. In addition, regressing zooids are full of hemocytes that make organ recognition difficult in section and in vivo zooid observations dark and lacking clarity. It is possible, therefore, that previous studies (not based on serial sections) were unable to recognize the complex, leading to the claim that the heart is the last organ to be resorbed [48].

Our behavioral experiments confirmed the lack of response in adult zooids to mechanical stimulation of the oral siphon as soon as it closes [49]. We previously showed that the number of oral siphon primary sensory cells (peripheral sensory neurons) decreases approaching takeover and correlates with a reduced response to stimuli [7]. Since unresponsiveness at takeover is temporally associated with the first signs of neurodegeneration, we suggest that a mechanism of transneuronal degeneration, involving sensory neurons and the connected brain neurons, could be responsible for the lack of sensitivity [1]. Additional research is required to investigate whether cell death induction is anterograde (i.e., loss of synaptic inputs from peripheral neurons induces associated brain neuron death) or retrograde (i.e., loss of brain neurons receiving afferent inputs from sensory cells induces death in the latter). Indeed, we were able to recognize residual synapses until the terminal phases of degeneration, though their functionality remains unclear. It is notable that the heart remains the only active organ until the terminal takeover; its beating likely facilitates in the circulation of numerous hemocytes in reducing zooids, some of them (like phagocytes) very large [24]. Although ascidian heart beating is generated by a myogenic mechanism involving several pacemakers [76], innervation is believed to participate in its regulation [34,72,77].

### 4.2. Apoptosis, Necrosis, and Autophagy Are Displayed by the Degenerating Neural Complex

Our results show that at takeover, the neural complex components of *B. schlosseri*, the cerebral ganglion and the neural gland, show multiple modes of cell death unequivocally recognized as signs of apoptosis, necrosis and autophagy at the ultrastructural level. Although it may be possible that *B. schlosseri* executes the genes in a different way than humans and mice, our transcriptomic analyses individuated some specific cell death sub-pathways, such as necroptosis and lysosomal cell death (considered different forms of necrosis) that can be discriminated only on the base of molecular/biochemical data. As also reported in mammals [1], many of the differentially expressed genes we found in this study belong to multiple forms of cell death, whereas some are typical of a specific cell death.

Typical apoptotic cytological alterations occur in the neural complex, as shown previously using TEM observations on other organs (branchial stigmata, stomach and intestine) [9,49] and using immunoblot and immunocytochemical data that provided evidence of both intrinsic and extrinsic pathways [78]. Indeed, the neural complex cells exhibit chromatin condensation at the nucleus periphery and nuclei that progressively lose their regular rounded/ovoid profile, becoming lobed. Cell cytoplasm maintains its electron density, meaning that ribosomes are not rarefied. From a transcriptomic point of view, we found several genes involved in apoptosis inhibition differently expressed in both the adults and in the buds. Particularly important is the greater expression of the apoptosis inhibitors *API5* and *SIX1* in the buds during takeover and in the filter-feeding adults compared with adults during takeover. Indeed, in humans and mice, the loss of *SIX1* promotes apoptosis through the inactivation of pro-apoptotic proteins such as caspases [53,79]. Moreover, a previous study on *B. schlosseri* showed their high expression in the entire colony during takeover compared with a colony in mid-cycle [80] and a greater amount of cell death during takeover [9,15], confirming the key role of *API5* and *SIX1* during takeover.

Surprisingly, we found that apoptosis was not the only form of cell death exhibited by the neural complex. Unequivocal signs of both necrosis and autophagy were frequently found in cells of the cerebral ganglion and the neural gland, both in its ciliated duct and in the gland body. Necrotic neurons, with swollen cytoplasm, were interspersed among apoptotic neurons. Moreover, neurons displayed numerous autophagic figures (autophagosomes delimited by a double membrane, autolysosomes with single membrane and electron-dense degraded content, and electron-dense degradative multilamellar bodies). The involvement of necrosis and autophagy in cell death was confirmed by the analyses of transcriptomes. *CTSD*, a key gene in the lysosomal cell death pathway, is highly expressed in the adult in takeover compared with the filter-feeding adult and the bud at takeover (Figure 7B). This trend supports the role of the cathepsins that can be released into the cytosol and initiate the lysosomal pathway of apoptosis through the cleavage of BID and the degradation of the anti-apoptotic Bcl-2 homologues [81]. This result is also supported by the downregulation of anti-apoptotic Bcl-2 during takeover [11]. Between the 25 BIRC genes that *B. schlosseri* has in its genome [82], we found that *BIRC3*, a gene involved both in the programmed cell death and in the necrosis pathways has an antiapoptotic role [83], is more expressed in filter-feeding adults as compared to adults during takeover, and more expressed in adults during takeover as compared to bud (Figure 7B). These results support previous studies on the whole colony transcriptome [80,82] that show diminished expression of *BIRC* during takeover. The reduced expression of this gene in the bud could indicate the absence of a need to suppress apoptosis. AMBRA is a positive regulator of autophagy. Its greater expression in the filter-feeding adults as compared to the adults in takeover is in accordance with previous investigations [12] and may suggest a dual role for this protein, as involved in both the developmental process and in the regulation of apoptosis.

Necrosis and autophagy were identified as providing a minor contribution to gut involution at takeover in *B. schlosseri* [49,84]. However, the evidence of multiple neuron death mechanisms is relevant in the parallels of neurodegeneration between this species and humans. Indeed, some human neurodegenerative diseases display the contemporary presence of different cell death mechanisms [1]. With the ambiguities displayed by some cell death pathways, future studies in *B. schlosseri* will be required to obtain causal evidence of the pathways here recognized, ideally using markers that appear exclusively in discrete forms of cell death. Indeed, the neuronal cell pathways found here may be identified on the basis of specific biochemical markers that will have to be tested (also in combination with other markers, in the absence of specific probes for *B. schlosseri*) to ascertain when and where these cell death mechanisms are involved in neurodegeneration.

Our data show that morula cells and phagocytes are involved in neurodegeneration at takeover, as they clearly infiltrate into (or are in strict contact with) the cerebral ganglion. Morula cells are the mediators of the inflammatory (rejection) reaction that occurs in the form of a series of necrotic spots along the contacting borders when genetically incompatible colonies are juxtaposed [43,85,86]. In both vertebrates and invertebrates, phagocytes are known to recognize dying cells as corpses and ingest them [78] thanks to a variety of “eat-me” signals, such as phosphatidylserine [87]. In *B. schlosseri*, this signal has been reported to be over-expressed in the dying hemocytes during takeover [84]. The number of these hemocytes changes significantly along with the blastogenic cycle [43,88]: comparing colonies in mid-cycle with colonies during takeover, morula cells increase from 40% to 60% of circulating hemocytes, whereas phagocytes rise from 4–10% to 20–30%. Future analysis to identify an "eat me” signal on the neurons’ surface will clarify the mechanism by which the phagocytes recognize them.

Morula cells enhance phagocytosis, releasing immunomodulatory factors (cytokines) [89] and expressing complement components influencing phagocytes [90,91]. Considering the role of immunocytes, and that all the differentially expressed transcripts here analyzed are involved in human inflammation (Table S1), future studies could consider if neurodegeneration in *B. schlosseri* works under the umbrella of “neuroinflammation”, which includes human glial (microglial and astrocyte) activation, typically associated with aging and many neurodegenerative disease [92]. Moreover, it is important to note that both morula cells and phagocytes in *B. schlosseri* participate in functional/physiological amyloidogenesis [93]; in human neurodegenerative diseases and during development, amyloidogenesis dysregulation has been shown to increase axonal removal by means of microglia and astrocytes [94,95,96].

### 4.3. A Conformational Disorder-Like Process Characterizes Neurodegeneration in B. schlosseri

Our data show that neurodegeneration in *B. schlosseri* presents both morphological and molecular manifestations also characteristic of human conformational disorders. In the latter, an unusual misfolding of proteins causes protein depositions in aggregates, triggering neuronal death [1,61,62,97,98]. These disorders include Alzheimer’s disease (tau and amyloid), Parkinson’s disease (-synuclein), Huntington’s disease (huntingtin), frontotemporal dementia (FTLD-Tau), and prion diseases (prion protein), whose molecular signatures were found to be increasing in *B. schlosseri* in late-cycle before takeover (present data) [7].

Among genes associated with prion diseases, the over-expression of some (PSEN1, FYN, SMC3, CHD2, EP300) in late-cycle could be rationalized by considering related proteins in humans, which, when expressed in their monomeric (soluble) form, exert protective functions for cellular homeostasis. In such cases, neurodegeneration is likely triggered by the progressive lack of monomers rather than by the aggregation itself [99]. This phenomenon may account for the lack of significant and long-lasting clinical benefits in parkinsonian patients undergoing monoclonal antibody therapy directed against aggregated alpha-synuclein [100,101]. Following this logic, the PSEN1 gene is of key importance, as it represents an overlapping gene between prion diseases and neurodegenerative disorders, including Alzheimer’s disease [61]. Moreover, it is noteworthy that an orthologue of the human amyloid precursor protein (APP) was identified in the solitary ascidian *Ciona intestinalis*: transgenic larvae expressing a human APP variant that form amyloid-like plaques in their nervous system and alter their behavior during settlement [102].

In this study, we observed (1) a progressive accumulation in *B. schlosseri* neurites of small vesicles (also elongated), with a diameter ranging from 25 to 100 nm, closely resembling the tubulovesicular structures considered the ultrastructural hallmark of prion diseases [64,65]. The tubulovesicular structures described previously are spheres or short rods of approximately 27 nm in diameter, although larger vesicular profiles measuring 100–110 nm in diameter have been reported, which would be comparable to those we found in *B. schlosseri*; (2) these small vesicles were found in neurites, but also in neural gland cells at terminal takeover, suggesting the progressive degenerative involvement of contiguous structures (from the ganglion to the gland) as occur in prion-like diseases over the course of many neurodegenerative disorders (such as Parkinson and Alzheimer’s diseases), both in human and animal models [61,103,104,105,106,107,108]; (3) a progressive medulla vacuolization that recalls the typical vacuolization of prion diseases characterized by a spongiform neuropil [66]; (4) both morphological and transcriptomic evidence of multiple neuronal cell death modes (apoptosis, necroptosis, lysosomal cell death, autophagy) that are characteristic of the conformational disorders referenced above; (5) 13 genes associated with prion diseases that are highly expressed just before the takeover onset, when the neural loss has just begun [7]. Therefore, we reason that protein aggregates can cause neuronal dysfunction and death, possibly through a cell-to-cell, prion-like propagation as suggested by both morphological findings and gene heatmaps. Although in the future, specific studies will be necessary to determine if misfolded proteins are the cause of neurodegeneration in *B. schlosseri*, this colonial chordate continues to demonstrate its value as a promising model for translational studies on human neurodegenerative diseases.

## Figures and Tables

**Table 1:** Description of main takeover events, which last about 48 h and repeat every week at 18 °C [44,48]).

Sub-Stage	Duration	Description
11^1^	2–4 h	Onset of takeover, recognizable by adult zooid siphon retraction and closure
11^2^	4–7 h	General shrinkage of regressing zooids that progressively reduce their dimension
11^3^	12–16 h	Further contraction of regressing zooids that in length are about half of their primary buds; inner organs, other than the beating heart, are no longer recognizable; zooids converge progressively toward the system center, in ventral position with respect to their growing primary buds
11^4^	6–9 h	Regressing zooids are spherical dark masses at the system center; their hearts do not beat

## Data Availability

The sequencing data analyzed in this study were published in [29] and are available in the NCBI Sequence Read Archive under accession: PRJNA579844.

## Supplementary Materials

The following supporting information can be downloaded at: https://www.mdpi.com/article/10.3390/cells12071041/s1, Figure S1: Main phases of the blastogenetic cycle of *B. schlosseri* and 3D reconstructions of the neural complex; Figure S2: Histology of the neural complex in a regressing zooid at sub-stage 11^1^; Figure S3: Histology of the neural complex in a regressing zooid at sub-stage 11^2^–11^3^; Figure S4: Histology of the neural complex in a regressing zooid at sub-stage 11^3^; Figure S5: Histology of the neural complex in a late primary bud; Figure S6: Histology of the neural complex in a regressing zooid at sub-stage 11^4^; Figure S7: Ultrastructure of the neural complex and adjacent tissues in a regressing zooid at sub-stage 11^1^; Table S1: List of genes involved in human neuronal death; Table S2: List of genes involved in human prion disease; Table S3: Differentially expressed genes associated with neuron death pathway between zooids in late cycle and takeover; Table S4: Differentially expressed genes associated with neuron death pathway between zooids and buds in takeover; Table S5: Relevant genes involved in neuronal death differentially expressed in the filter-feeding adults vs. adults in takeover and in adults in takeover vs. bud.
