# Supplementary material for: Multiple Forms of Neural Cell Death in the Cyclical Brain Degeneration of A Colonial Chordate
Source: Cells. 2023 Mar 29;12(7):1041. doi: 10.3390/cells12071041 (PMC10093557; doi:10.3390/cells12071041)
Supplement: Supplementary file 1 [file cells-12-01041-s001.zip › cells-2274665-supplementary.pdf]

Supplementary Materials

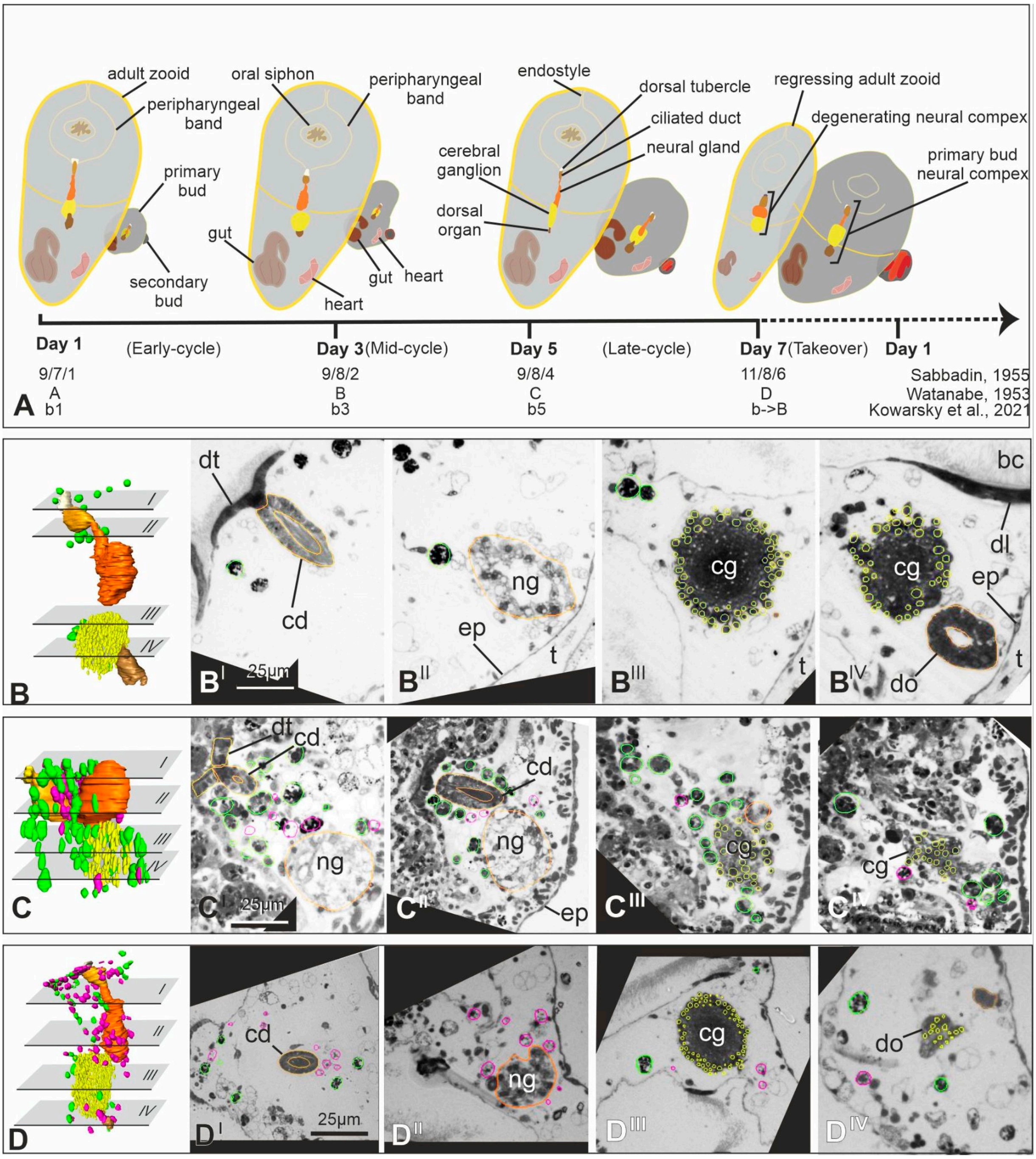

Figure S1. A. Main phases of the blastogenetic cycle of *B. schlosseri*; for simplicity, only an adult zooid and its right primary bud (with its own secondary bud) is shown. The takeover occurs at day 7. Note that, at takeover, whereas the neural complex is degenerating in the regressing adult zooid, in the primary bud it is refining its configuration to become fully functioning the day after (see Fig. 1E-F). 9/7/1, 9/8/2, 9/8/4, and 11/8/6 refer to the colony phases introduced by Sabbadin, 1955; A, B, C, and D to the secondary bud stages introduced by Watanabe (1953); b1, b3, b5, and b->B to the secondary bud stages introduced by Kowarsky and collaborators (Kowarsky et al.,

2021). **B-D.** 3D reconstructions of the neural complex (see Fig. 1D-F for color codes) and transverse serial histological sections (Toluidine blue), from posterior to anterior, of the neural complex of a filter-feeding adult (mid-cycle, phase 9/8/3, B^{I}-B^{IV}), and adult in takeover (11^2/8/6, C^{I}-C^{IV}), and a primary bud at takeover (phase 9/8/6, D^{I}-D^{IV}). B and C show the neural complex structures segmented on the adjacent sections. The cerebral ganglion (cg) is ventral to the neural gland (ng), however in takeover, because of zooid shrinkage, the relationships between the two organs are not maintained. Scale bar is the same in B and in C. bc: branchial chamber; cd: ciliated duct; cg: cerebral ganglion; dl: dorsal lamina; do: dorsal organ; dt: dorsal tubercle; ep: epidermis; ng: neural gland; t: tunic.

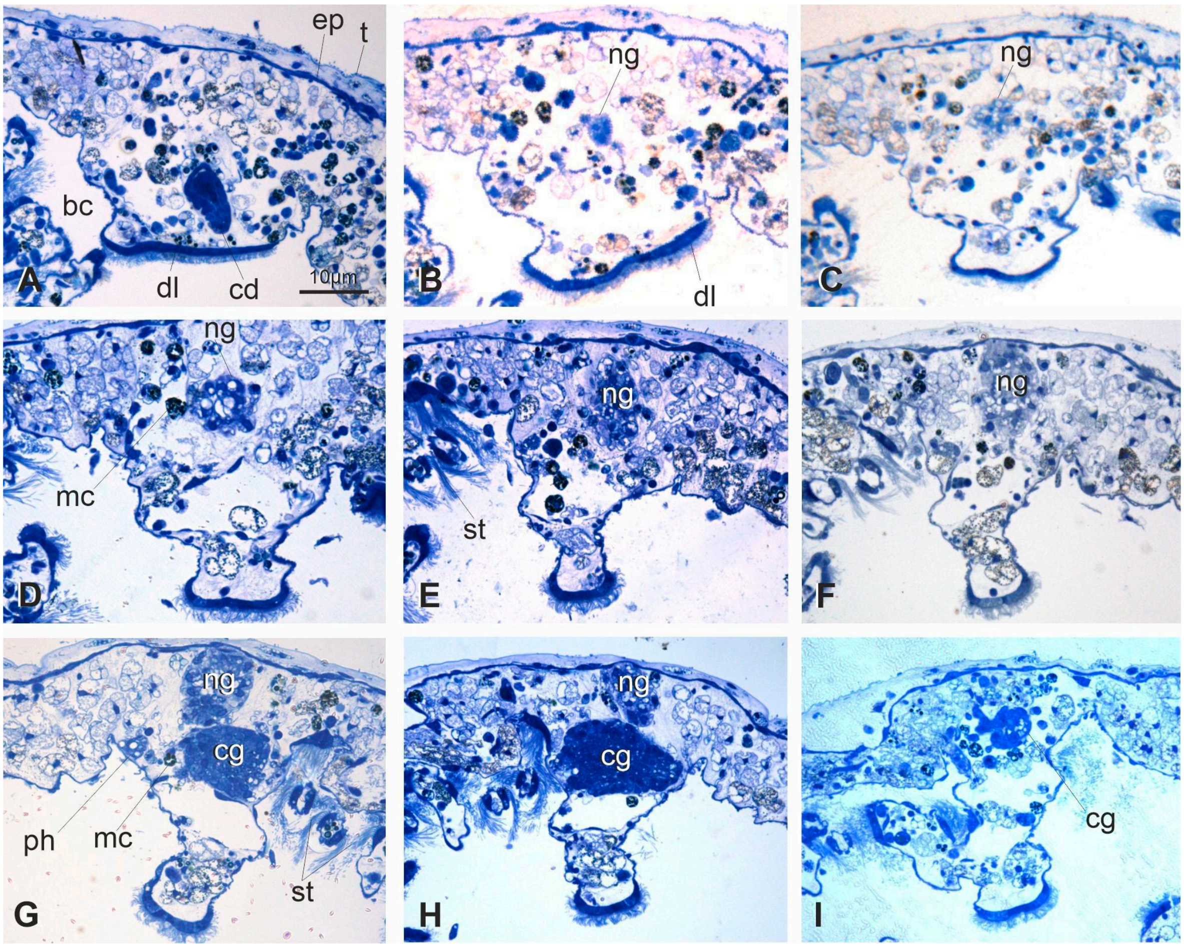

Figure S2 A-I. Histology of the neural complex in a regressing zooid at sub-stage 11^1, at neural complex level. Note that the dorsal lamina (dl) possesses cells with different labeling affinity, as a sign of initial degeneration. Cross sections from anterior to posterior (selected from a complete series. Dorsal at top. Toluidine blue. bc: branchial chamber; cd: ciliated duct of the neural gland; cg: cerebral ganglion; dl: dorsal lamina; ep: epidermis; mc: morula cell; ng: neural gland; ph: phagocyte; st: stigmata; t: tunic.

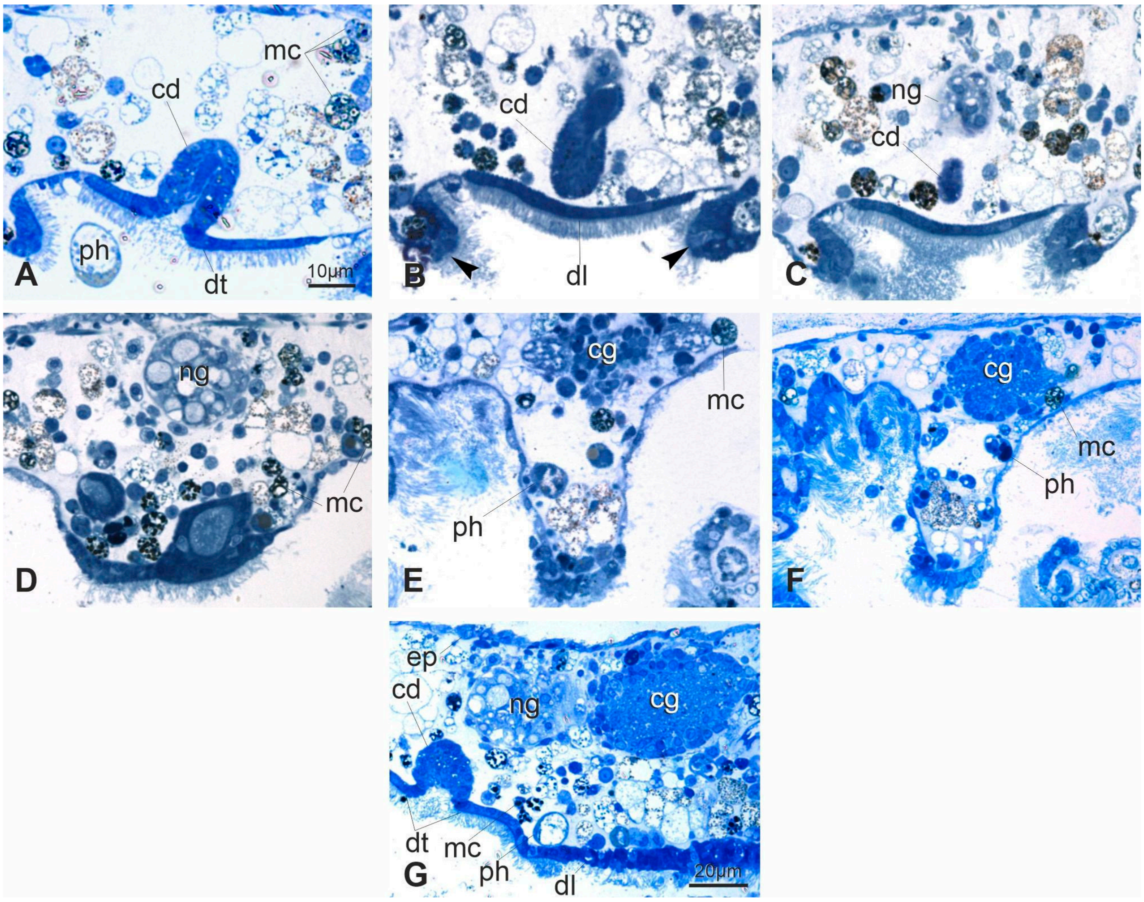

Figure S3. Histology of the neural complex in a regressing zooid at sub-stage 11^2-11^3. Toluidine blue. **A-F**. Cross sections from anterior to posterior (selected from a complete series). Dorsal at top. **G**. Sagittal medial section. Arrowheads in B: peripharyngeal band; cd: ciliated duct of the neural gland; cg: cerebral ganglion; dl: dorsal lamina; dt: dorsal tubercle; ep: epidermis; mc: morula cell; ng: neural gland; ph: phagocyte.

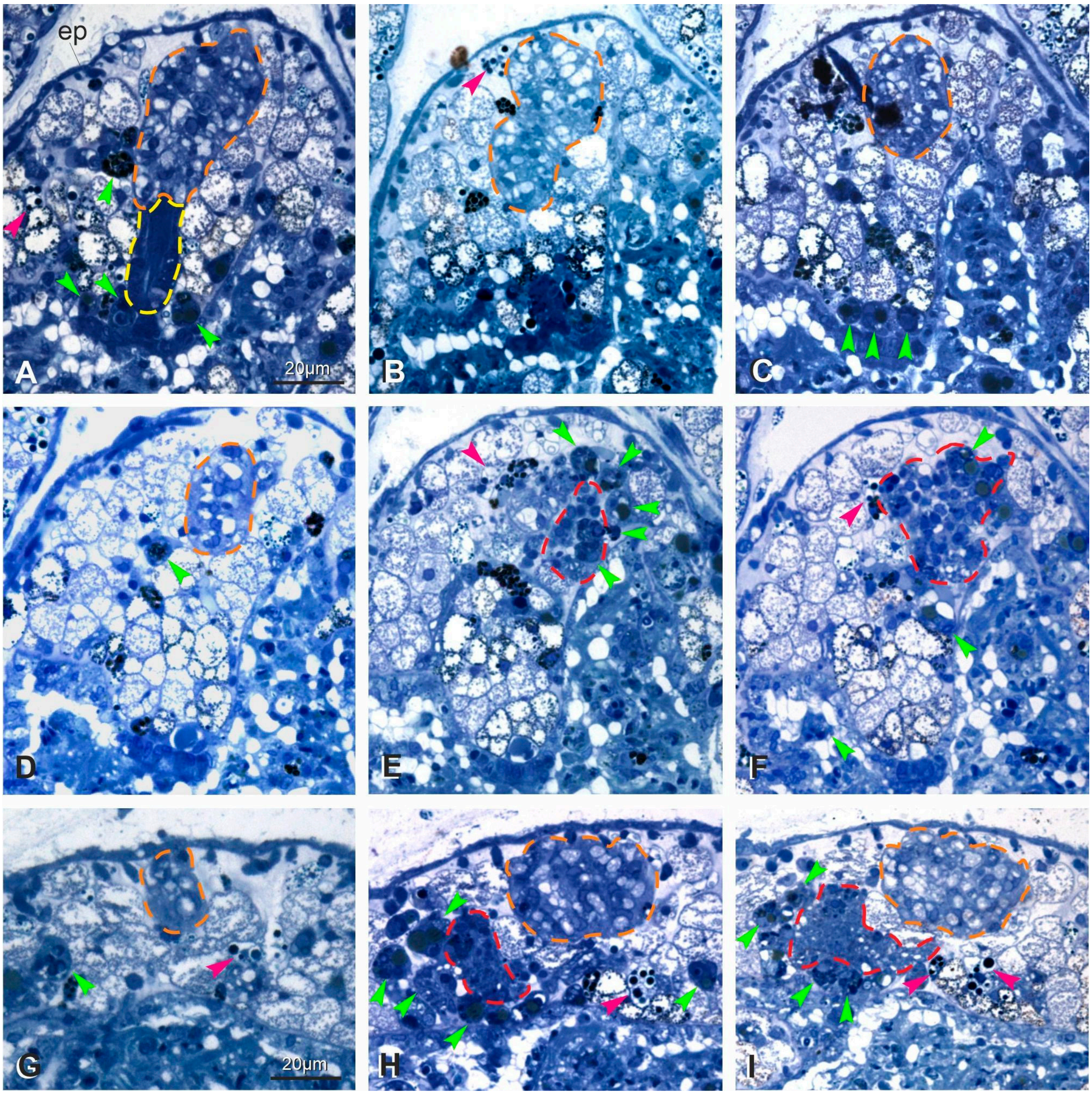

Figure S4. Histology of the neural complex in a regressing zooid at sub-stage 11^{3}. Toluidine blue. **A-F**. Cross sections from anterior to posterior (selected from a complete series). Dorsal at top. **G-I**. Sagittal medial sections from right to left (selected from a complete series). Dorsal at top; anterior at left. Orange dotted line: neural gland; red dotted line; cerebral ganglion; yellow dotted line: ciliated duct of the neural gland; pink arrowheads: morula cells; green arrowheads: phagocytes. ep: epidermis.

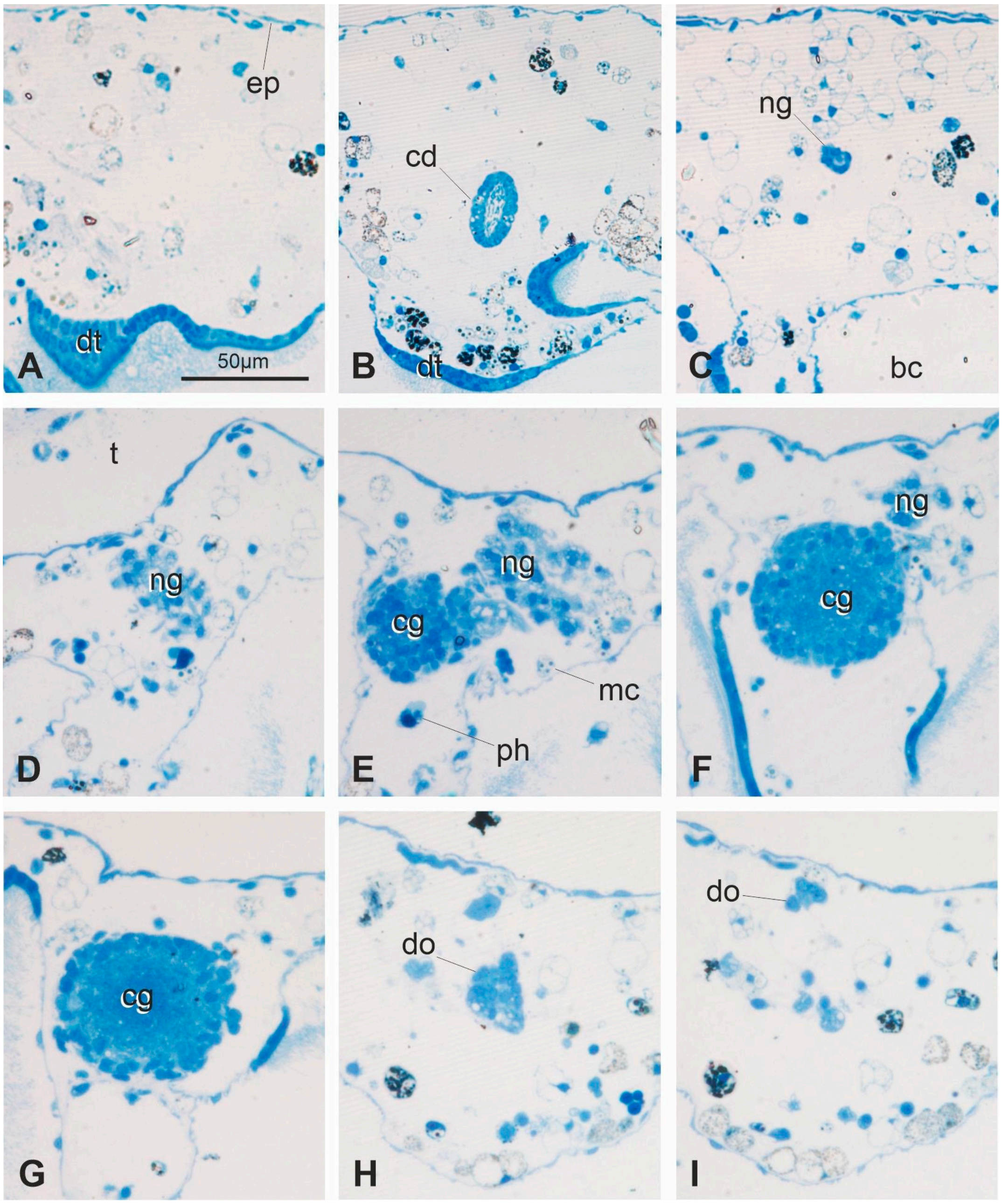

Figure S5 A-I. Histology of the neural complex in a late primary bud, just before the takeover. Cross sections from anterior to posterior (selected from a complete series). Dorsal at top. Toluidine blue. bc: branchial chamber; cd: ciliated duct of the neural gland; cg: cerebral ganglion; dl: dorsal lamina; do: dorsal organ; dt: dorsal tubercle; ep: epidermis; mc: morula cell; ng: neural gland; ph: phagocyte; t: tunic.

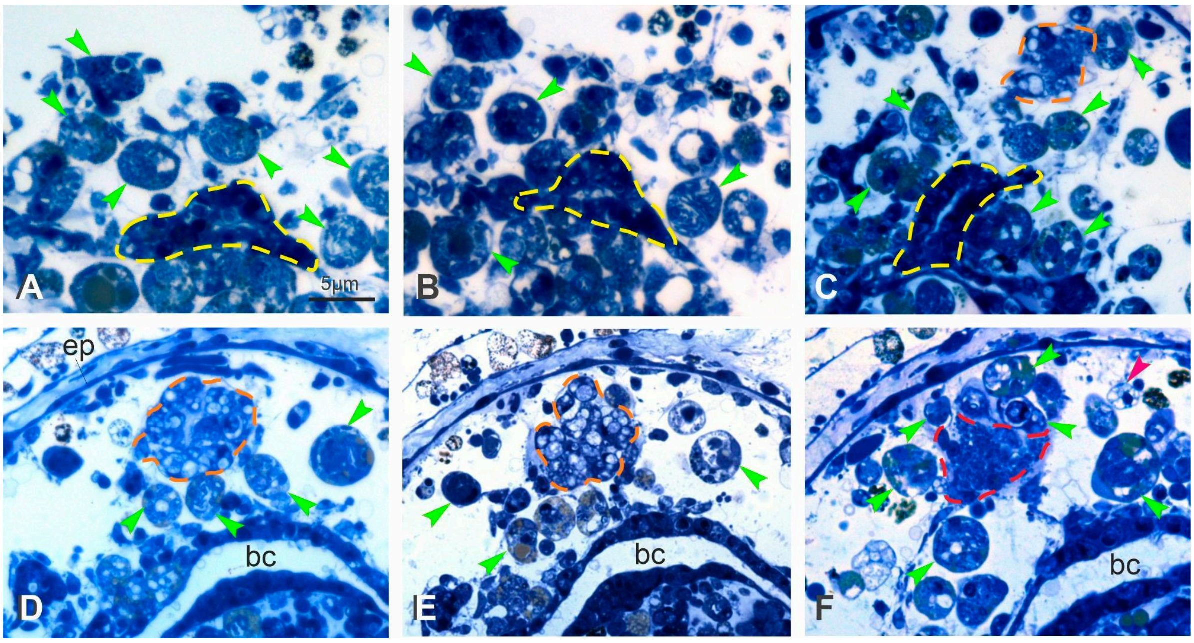

Figure S6 A-F. Histology of the neural complex in a regressing zooid at sub-stage 11^4. Cross sections from anterior to posterior (selected from a complete series). Dorsal at top. Toluidine blue. Orange dotted line: neural gland; red dotted line; cerebral ganglion; yellow dotted line: ciliated duct of the neural gland; pink arrowheads: morula cells; green arrowheads: phagocytes. bc: branchial chamber; ep: epidermis.

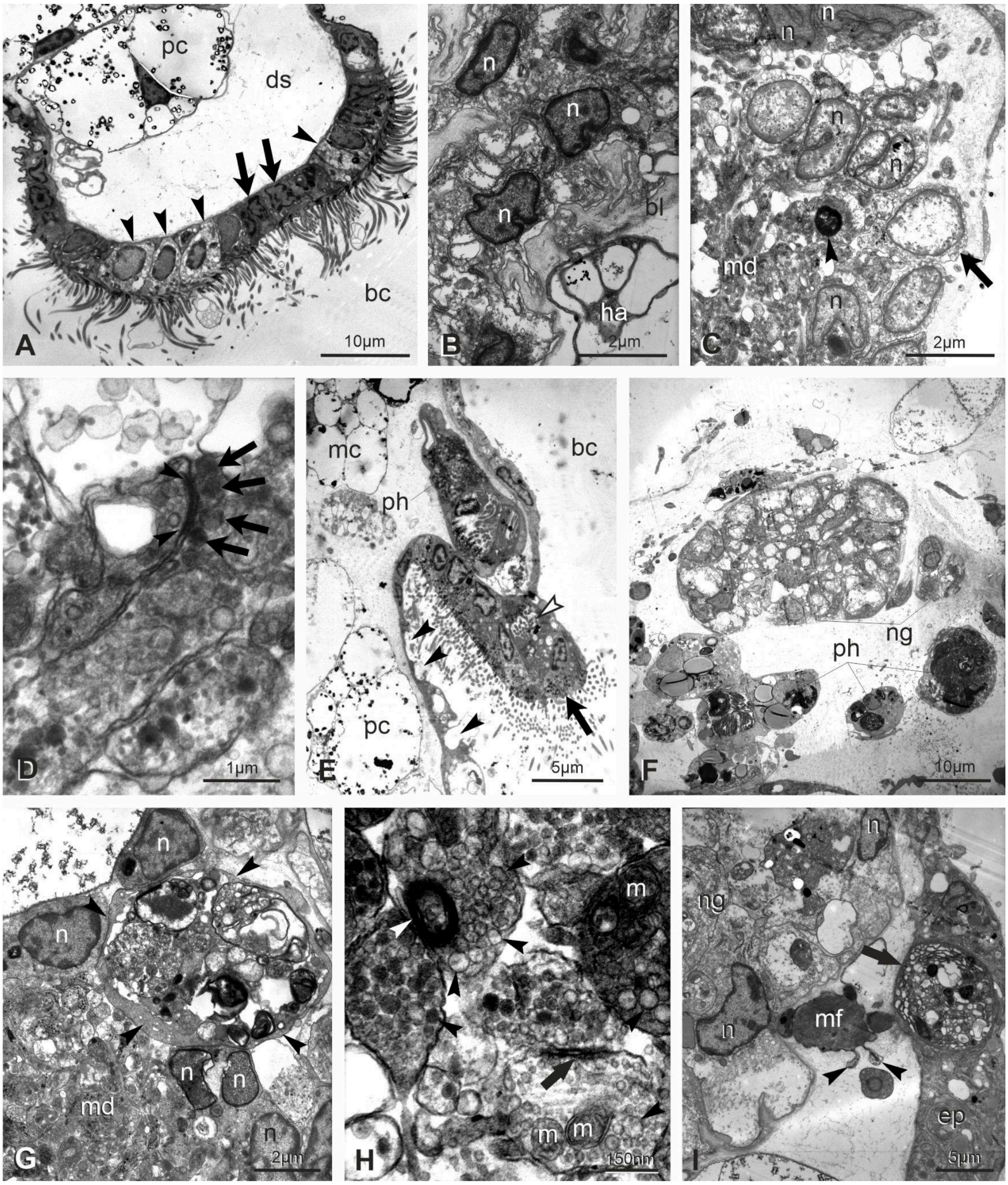

Figure S7. Ultrastructure of the neural complex and adjacent tissues in a regressing zooid at sub-stage 11[1]. TEM. **A.** Detail of dorsal lamina in cross section. Some cells (arrowheads) are swollen; other cells (arrows) have nuclei with irregular profile and chromatin condensed at the nucleus periphery. bc: branchial chamber; ds: dorsal sinus lumen occupied by a large pigmented cell (pc). **B.** Hyaline amebocyte (phagocyte precursor) (ha) close to the neural gland cells. bl: neural gland basal lamina; n: neural gland cell nucleus. **C.** Neurons in the cerebral ganglion cortex. Some of them have lobed nuclei (n). The medulla (md) is not tightly packed. Arrow: swollen neuron; arrowhead: degraded authophagic vacuole. **D.** A synapsis in the cerebral ganglion medulla, showing presynaptic vesicles (arrows) and postsynaptic densities (arrowheads). **E.** A morula cell (mc) and a phagocyte (ph) close to the peripharyngeal band (arrow). The phagocyte possesses phagosomes with remnants of ciliated cells. The adjacent epithelium of the

peripharyngeal band presents lobed nuclei with chromatin condensed at the periphery and large apical cell membrane invaginations (black arrowheads). The white arrowhead points at a cell invagination cut tangentially, in which some outer cilia are recognizable. bc: branchial chamber; pc: pigmented cell. **F.** Numerous large phagocytes (ph) close to the neural gland (ng). Note the heterogeneous content of their phagosomes. **G.** A large phagocyte (arrowheads) infiltrating the cerebral ganglion. md: medulla; n: neuron nucleus. **H.** Morphological alterations in neurites: note the number of small vesicles (black arrowheads) filling neurites. A synapsis (arrow) and a degradative multilamellarbody (white arrowhead) are recognizable. m: mitochondrion. **I.** A phagocyte (arrow) infiltrating the epidermis (ep) close to the neural gland (ng). Note that the muscle fiber (mf) shows signs of alterations as membrane blebbing (arrowheads). n: nucleus of a neural gland cell.
